# Single low-dose INC280-loaded theranostic nanoparticles achieve multirooted delivery for MET-targeted primary and liver metastatic NSCLC

**DOI:** 10.1186/s12943-022-01681-y

**Published:** 2022-12-01

**Authors:** Yige Sun, Jie Yang, Yingbo Li, Jing Luo, Jiemei Sun, Daoshuang Li, Yuchen Wang, Kai Wang, Lili Yang, Lina Wu, Xilin Sun

**Affiliations:** 1 Department of Nuclear Medicine, the Fourth Hospital of Harbin Medical University, Harbin, 150028 Heilongjiang China; 2 NHC Key Laboratory of Molecular Probe and Targeted Diagnosis and Therapy, Molecular Imaging Research Center (MIRC), Harbin Medical University, Harbin, 150028 Heilongjiang China

**Keywords:** NSCLC, Liver metastasis, Capmatinib, Perfluorocarbon, Pulmonary delivery, ^19^F-MRI, Single low-dose

## Abstract

**Background:**

Non-small cell lung cancer (NSCLC) patients with primary tumors and liver metastases have substantially reduced survival. Since mesenchymal-epithelial transition factor (MET) plays a significant role in the molecular mechanisms of advanced NSCLC, small molecule MET inhibitor capmatinib (INC280) hold promise for clinically NSCLC treatment. However, the major obstacles of MET-targeted therapy are poor drug solubility and off-tumor effects, even oral high-dosing regimens cannot significantly increase the therapeutic drug concentration in primary and metastatic NSCLC.

**Methods:**

We developed a multirooted delivery system INC280-PFCE nanoparticles (NPs) by loading INC280 into perfluoro-15-crown-5-ether for improving MET-targeted therapy. Biodistribution and anti-MET/antimetastatic effects of NPs were validated in orthotopic NSCLC and NSCLC liver metastasis models in a single low-dose. The efficacy of INC280-PFCE NPs was also explored in human NSCLC specimens.

**Results:**

INC280-PFCE NPs exhibited excellent antitumor ability in vitro. In orthotopic NSCLC models, sustained release and prolonged retention behaviors of INC280-PFCE NPs within tumors could be visualized in real-time by ^19^F magnetic resonance imaging (^19^F-MRI), and single pulmonary administration of NPs showed more significant tumor growth inhibition than oral administration of free INC280 at a tenfold higher dose. Furthermore, a single low-dose INC280-PFCE NPs administered intravenously suppressed widespread dissemination of liver metastasis without systemic toxicity. Finally, we verified the clinical translation potential of INC280-PFCE NPs in human NSCLC specimens.

**Conclusions:**

These results demonstrated high anti-MET/antimetastatic efficacies, real-time MRI visualization and high biocompatibility of NPs after a single low-dose.

**Supplementary Information:**

The online version contains supplementary material available at 10.1186/s12943-022-01681-y.

## Introduction

Non-small cell lung cancer (NSCLC) treatment remains a great challenge, as more than 60% of patients are diagnosed with locally advanced or metastatic stages [1]. The liver is a prevalent site of metastasis in NSCLC because of its abundant blood supply, causing rapid invasion of tumor cells [2]. Aberrant activation of mesenchymal-epithelial transition factor (MET) causes c-MET dimerization, autophosphorylation, and kinase activity that are essential for malignant transformation, facilitating tumor progression and metastasis [3–6]. Recently, MET-targeted therapy of advanced NSCLC with driver mutations has been recommended and has thus entered a stage of standardization [7]. On Aug 10, 2022, the orally available compound capmatinib (INC280) obtained regular approval of Food and Drug Administration (FDA) for advanced/metastatic NSCLC patients with MET dysregulation [8]. Although promising, the clinical utility of oral INC280 is restricted by its strong hydrophobicity, low bioavailability, short half-period and distribution to nontargeted sites [9]. Repeated administration of INC280 inevitably causes adverse reactions and systemic toxicity, including peripheral edema, hepatotoxicity, nephrotoxicity, nausea and vomiting [10]. More importantly, the cost of the recommended schedule of INC280 is quite high, making this regimen unattainable for many patients. Consequently, more efficient therapeutics are desirable to improve the anti-MET/antimetastatic efficacies of INC280 in clinical practice.

Nanoformulations have measurable clinical translation potential to elevate the efficacy and biosafety of antineoplastic drugs [11]. Perfluorocarbon nanoparticles (PFC NPs) are optimal delivery vectors that can be easily functionalized with therapeutic agents to improve drug solubility and pharmacokinetics [12]. In particular, perfluoro-15-crown-5-ether (PFCE) NPs remain relatively long and stable bioretention and extremely favorable ^19^F magnetic resonance imaging (^19^F-MRI) properties, which provides an opportunity for the construction of more effective image-guided therapeutic platform for precise cancer treatment [13–17]. In view of their high oxygen-carrying capacity and good biocompatibility, direct pulmonary delivery of PFCE NPs offers a promising approach against primary NSCLC [18, 19]. Our previous study demonstrated that pulmonary administration of PFCE NPs resulted in high drug concentrations in lung cancer tissues and that could be quantitatively imaged by ^19^F-MRI with minimal extratumoral systemic exposure [20]. In addition, following intravenous (IV) injection, PFCE NPs can be rapidly and steadily trapped in the liver, which holds promise for the treatment of liver metastases. However, there have been no reports on their application.

Currently, there are few studies on PFCE NPs loaded with drugs, especially small molecule inhibitors, due to the strong hydrophobicity and rapid blood clearance of the drugs. It is therefore necessary to conduct an intensive study of the potential of the PFCE NP platform to increase drug penetration and allow sustained drug release. These properties will facilitate a reduction in the frequency of dosing and the received total doses, improving compliance and safety in clinical practice. Here, we developed a multirooted drug delivery system derived from PFCE NPs for delivering a small molecule MET inhibitor to tumors as an effective theranostic strategy against advanced/metastatic NSCLC. The present study for the first time considers the loading of INC280 into the PFCE NPs, which have been termed INC280-PFCE NPs. In our in vitro mechanism studies, INC280-PFCE NPs exhibited excellent antitumor activity via blockade of c-MET signaling pathways. In orthotopic NSCLC models, pulmonary delivery of single low-dose INC280-PFCE NPs altered the biodistribution of INC280, which effectively inhibited NSCLC tumor progression. In addition, IV injection of INC280-PFCE NPs represent a breakthrough in the treatment of NSCLC liver metastases due to their long-term retention in lesions and reduction in off-target and toxic effects, compared with oral administration of INC280. Finally, ex vivo study in human lung cancer specimens further demonstrated the clinical translation ability of INC280-PFCE NPs.

## Materials and methods

### Cell culture and animals

Human NSCLC cell line EBC-1 (MET amplification) was acquired from the Japanese Collection of Research Bioresources (JCRB) (Osaka, Japan). Minimum essential medium (MEM; Procell, China) for EBC-1 cell culture was supplemented with 10% fetal bovine serum (FBS) and 1% (v/v) penicillin/streptomycin. Female BALB/c nude mice (Charles River) were raised in a specific pathogen–free (SPF) and humidity-controlled room. All mice handling was in terms of the procedures approved by the Harbin Medical University Institutional Animal Care and Use Committee.

### *Orthotopic NSCLC model and *in vivo* MRI*

The orthotopic NSCLC mouse model is extensively used as a disease-related animal model in cancer research field, owing to it is close resemblance to human NSCLC, in terms of gene expression, molecular characteristics, histopathology and biology [21]. The orthotopic NSCLC model was established as previously described [22]. Briefly, mice were anesthetized with Zoletil™ 50 (25 μL). With the aid of mouse laryngoscopy, the main bronchi of the mice were administered with EBC-1 cell suspension (50 μL, 10^7^ cells/mL). When the tumors reached ~ 30 mm^3^ in size, the mice were subjected to one of the following treatments: INC280-PFCE NPs or PFCE NPs (50 µL, *n* = 3) by intratracheal (IT) administration. All imaging experiments were performed on a 9.4 T MR scanner with a ^1^H/^19^F dual-tuned volume coil. Images were acquired at 1 h to 7 days after administration. Throughout the imaging experiments, the mice were anesthetized by inhalation of isoflurane to keep the respiration rate between 60 ~ 90/minute. ^1^H-MR images were acquired by the axial T1-Rapid Acquisition with Relaxation Enhancement (RARE) sequence, repetition time (TR)/echo time (TE) = 820/12 ms, RARE factor = 8, number of averages (NA) = 8, slice thickness (ST) = 1 mm, matrix = 256 × 256, and field of view (FOV) = 38.4 × 38.4 mm^2^. The corresponding ^19^F-MRI were acquired by RARE sequence, TR/TE = 2000/100 ms, RARE factor = 32, NA = 128, ST = 3 mm, matrix = 64 × 64 and FOV = 38.4 × 38.4 mm^2^. The ^19^F-MR images were merged with the corresponding ^1^H-MR images to identify the exact anatomic location. For ^19^F-MRI quantification, PFCE concentration in tumors was calculated based on the known concentration of the reference phantom and the ^19^F-MR images signal-to-noise ratio (SNR). A reference phantom containing 20.74 mg/mL PFCE NPs was placed next to mice for quantification of ^19^F-MRI data. The formula was C_t_ = C_r_ × SNR_t_/SNR_r_ (C_t_ is the PFCE concentration in tumor, C_r_ is the PFCE concentration of reference phantom, SNR_t_ is the ^19^F-MR images SNR of the tumors, and SNR_r_ is the ^19^F-MR images SNR of the reference phantom).

### In vivo* therapeutic efficacy*

Orthotopic NSCLC models were established as described above (n = 13 per group). When tumors reached ~ 30 mm^3^ in size, female BALB/c nude mice were divided into 4 groups at random: Control, PFCE NPs, INC280 and INC280-PFCE NPs. INC280 was orally given at 3 mg/kg every day, whereas the INC280-PFCE NPs groups were administered a single 50 µL dose by IT instillation. The tumor volumes and body weights of the mice were monitored every two days. Tumor volumes were monitored by T1-weighted MRI. Survival was monitored to day 60 (*n* = 8). On day 14 posttreatment, the excised tumors from mice were weighed and photographed. The excised tumors and major organs were collected for histopathological analysis. Hematoxylin and eosin (H&E) staining, immunohistochemical (IHC) staining (phospho-MET, Ki67 and CD31) and TUNEL assays were performed to confirm the treatment effect. NSCLC liver metastasis models were established as previously described (*n* = 13 per group). The mice were assigned to four groups at random and administered PBS, PFCE NPs, INC280 and INC280-PFCE NPs. INC280 was orally given at 3 mg/kg every day, whereas the INC280-PFCE NPs groups were administered a single 100 µL dose by IV injection. Tumor volumes and the number of metastatic nodes were monitored by T2-weighted MRI every other day. Survival study and body weights record were consistent with previous descriptions. On day 14 posttreatment, the livers were weighed, photographed and performed with H&E staining and Ki67 staining as described in previous reports.

### Ex vivo* targeted treatment of human lung cancer specimens*

Human lung cancer specimens were collected from NSCLC patients who underwent surgery. All human specimens related studies were in accordance with the related ethical regulations of the ethics committee of the Fourth Affiliated Hospital of Harbin Medical University. Six patients in total were eligible for the research and signed informed consent for usage of medical history data and biospecimens. Tumor tissues were processed and cultured as previously described [23, 24]. Briefly, freshly surgically resected human lung cancer tissues were immediately washed with DMEM (high glucose) (LONSERA) containing 10% FBS. Then, the tumor tissues were divided into 9 equal parts with a scalpel under sterile conditions, each of which was 40 ~ 50 mm^3^ in size. Each tumor fragment was incubated in a 12-well culture plate containing medium with PBS, INC280 or INC280-PFCE NPs. The groups of INC280 and INC280-PFCE NPs had the same concentration of INC280 (10 nM). After 48 h of ex vivo incubation, the therapeutic effect of each treatment on the patient-derived tumor fragments (PDTFs) was verified by western blot, H&E staining and IHC assays (total MET and phospho-MET).

### Statistical analysis

All statistical analyses were carried out using GraphPad Prism 8.0. Statistical evaluation was performed with the Student’s t test and one- or two- way ANOVA. All data are represented as the means ± standard deviation (SD), and *P* < 0.05 represents statistical significance.

## Results

### Synthesis and characterization of INC280-PFCE NPs

**Fig. 1:**
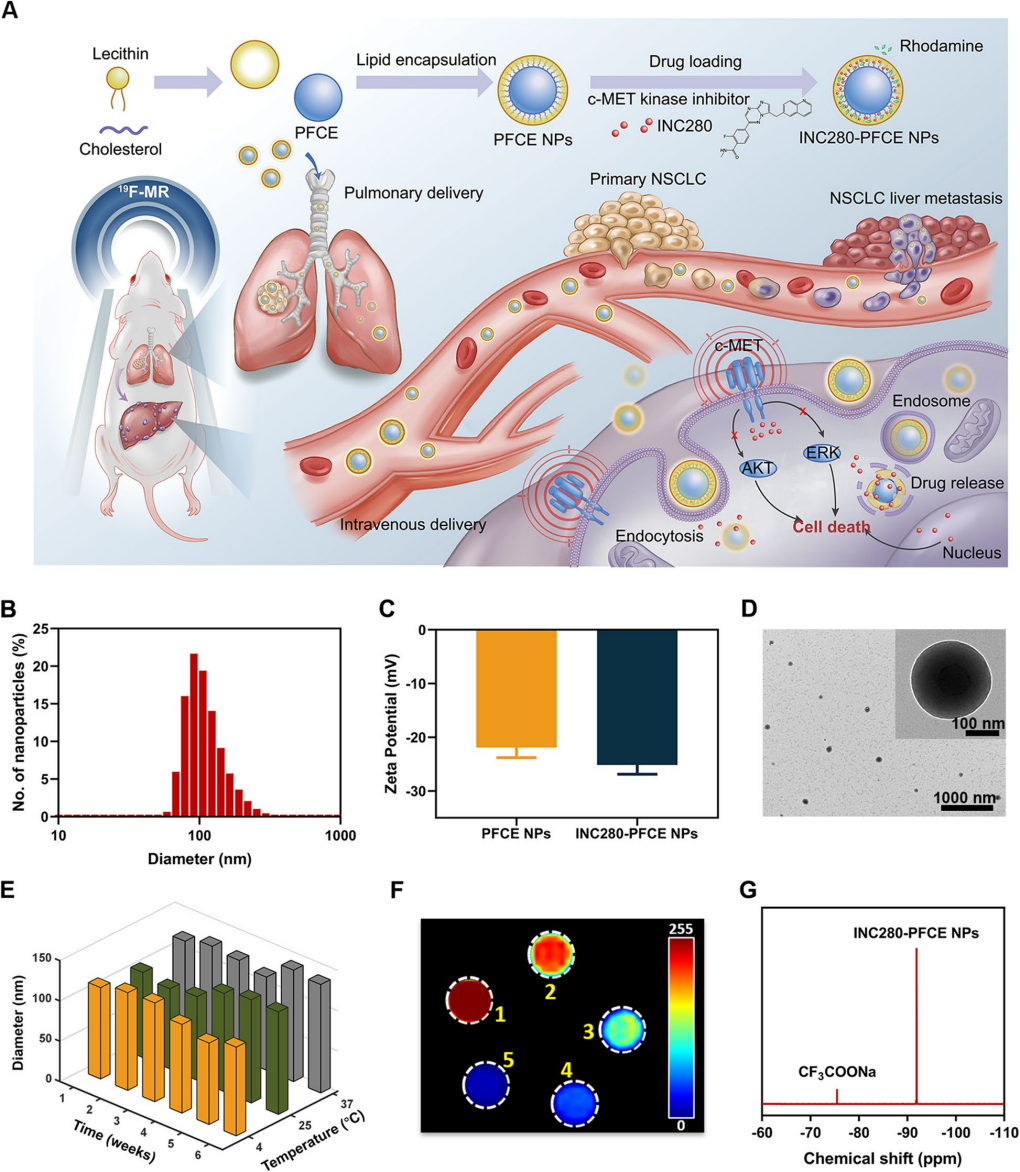
Schematic illustrations of synthesis of INC280-PFCE NPs and corresponding theranostic strategy and characterization. **A** Scheme of the synthesis of the INC280-PFCE NPs and theranostic strategy. **B** Size distribution of INC280-PFCE NPs determined by DLS. **C** The average zeta potential values of PFCE NPs and INC280-PFCE NPs. **D** INC280-PFCE NPs were characterized by TEM. Scale bar is 100 nm for small view and 1000 nm for large view. **E** After storage at 4, 25 and 37 °C, size stability tests of INC280-PFCE NPs over 6 consecutive weeks. **F** ^19^F-MR images of INC280-PFCE NPs with increasing concentrations of ^19^F (C_F_). 1–5: 2.59, 5.19, 10.37, 20.74, 41.48 mg/mL, respectively. **G** ^19^F-NMR of INC280-PFCE NPs

The synthesis of the INC280-PFCE NPs and corresponding theranostic strategy is illustrated schematically in Fig. 1A. INC280-PFCE NPs were prepared via a microfluidics process with high encapsulation efficiency (EE) of INC280 (89.75%). To optimize the hydrophobicity of INC280 and maximize the fluorine content in the INC280-PFCE NPs, natural phospholipids (lecithin) and cholesterol were used to prepare surfactant-lipid complexes, and INC280 was encapsulated into the lipid layer. Then, the as-prepared INC280-PFCE NPs and PFCE NPs were characterized by size distributions and zeta potentials. The INC280-PFCE NPs and PFCE NPs were 114.5 ± 5.83 nm and 83.1 ± 8.19 nm in diameter, respectively (Fig. 1B, Supplementary Fig. 1). The average zeta potential values of the INC280-PFCE NPs and PFCE NPs were negative (-25.2 mV and -21.9 mV, respectively) (Fig. 1C). The INC280-PFCE NPs displayed regular spherical structures as observed by transmission electron microscopy (TEM) (Fig. 1D, Supplementary Fig. 2). Moreover, the INC280-PFCE NPs in deionized water exhibited good stability after storage at 4 °C for up to 6 weeks. And the INC280-PFCE NPs’ size increased slightly at 25 °C and 37 °C, particularly after 5 to 6 weeks of storage (Fig. 1E, Supplementary Table 1). And there were no significant changes of diameter in FBS and culture medium solution for up to 14 day (Supplementary Fig. 3). The signal-to-noise ratio (SNR) of ^19^F-MRI was linearly related to increasing concentrations of the INC280-PFCE NPs, demonstrating their excellent ^19^F-MRI properties (Fig. 1F, Supplementary Fig. 4). The ^19^F NMR spectrum of the INC280-PFCE NPs exhibited a single fluorine peak at − 91.9 ppm with a CF_3_COONa peak at − 75.4 ppm as a reference (Fig. 1G).

### *INC280-PFCE NPs uptake efficiency and antitumor effect *in vitro

**Fig. 2:**
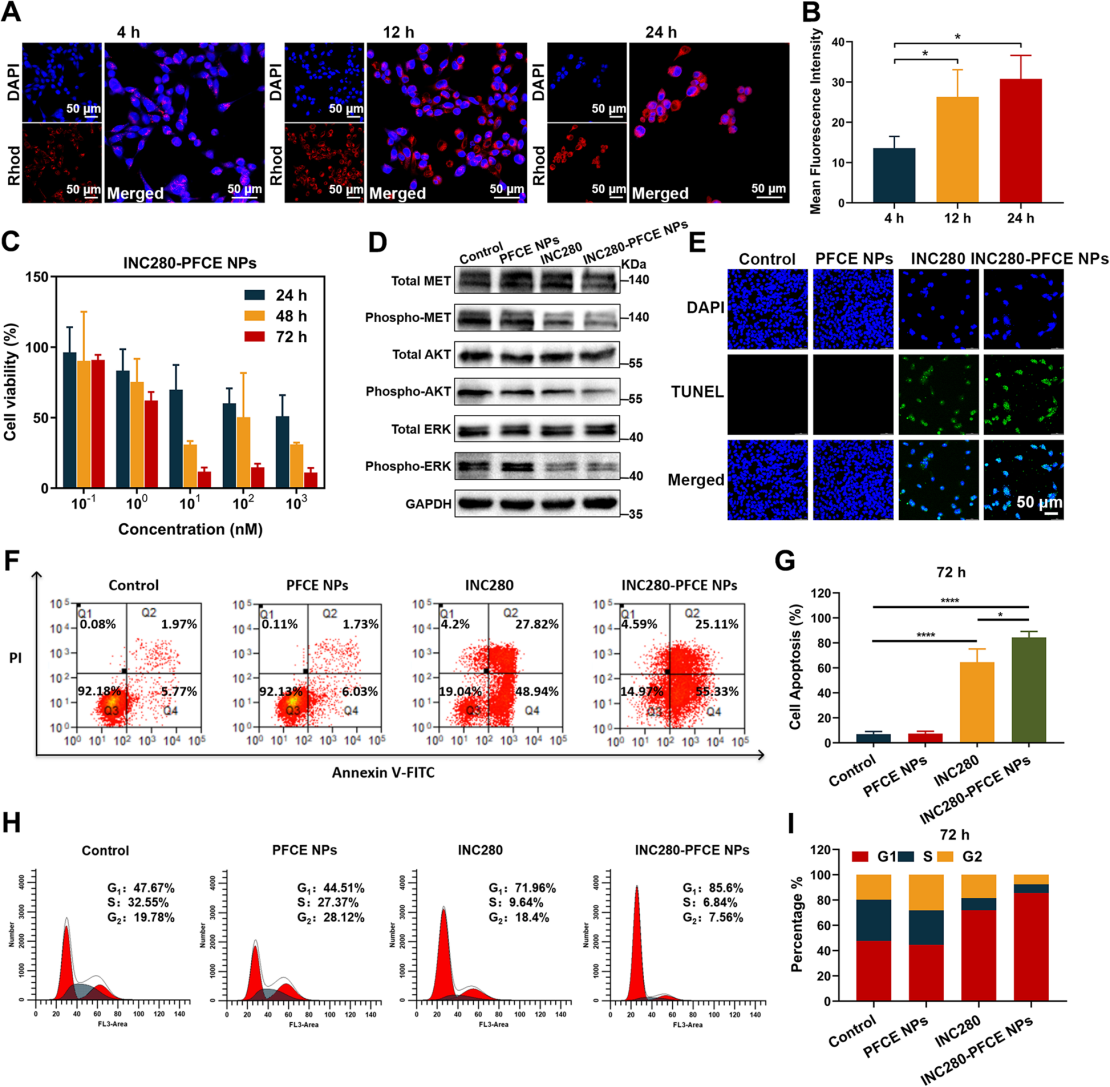
INC280-PFCE NPs uptake efficiency and antitumor effect in vitro. **A** Representative CLSM images. EBC-1 cells were incubated with rhodamine-labeled INC280-PFCE NPs (10 nM INC280) for 4 h, 12 h and 24 h (red). The nuclei were stained with DAPI (blue)**.** Scale bar is 50 µm. **B** After incubation for 4 h, 12 h and 24 h, analysis of the mean fluorescence intensity (MFI) of INC280-PFCE NPs in EBC-1 cells. **C** After incubation with different concentrations of INC280-PFCE NPs (10 nM INC280), viability of EBC-1 cells at 24, 48 and 72 h. **D** Western blot analysis of MET and its downstream signaling pathway proteins in EBC-1 cells incubated with different treatments for 4 h. The group included Control, PFCE NPs (30 nM PFCE), INC280 (1 nM INC280) and INC280-PFCE NPs (1 nM INC280 and 30 nM PFCE). **E** After 72 h of treatment with different formulations**,** detection of EBC-1 cell apoptosis by TUNEL assays. The group included Control, PFCE NPs (300 nM PFCE), INC280 (10 nM INC280) and INC280-PFCE NPs (10 nM INC280 and 300 nM PFCE). Scale bar = 50 µm. **F** After 72 h of treatment with different formulations, detection of EBC-1 cell apoptosis by flow cytometry. The group included Control, PFCE NPs (300 nM PFCE), INC280 (10 nM INC280) and INC280-PFCE NPs (10 nM INC280 and 300 nM PFCE). **G** Quantification of cell apoptosis analyzed by flow cytometry. **H** After 72 h of treatment with different formulations, cell cycle analyzed by flow cytometry. The group included Control, PFCE NPs (300 nM PFCE), INC280 (10 nM INC280) and INC280-PFCE NPs (10 nM INC280 and 300 nM PFCE). **I** Quantitative analysis of EBC-1 cells in G1, S and G2 phases. Data are shown as mean ± standard deviation (*n* = 3; * *P* < 0.05, **** *P* < 0.0001)

The images of confocal laser scanning microscopy (CLSM) revealed the uptake efficiency of INC280-PFCE NPs by EBC-1 cells (Fig. 2A). The fluorescence signal was detected at 4 h, and the uptake efficiency of the INC280-PFCE NPs increased significantly at 12 h and 24 h, indicating time-dependent uptake (Fig. 2B). In addition, PFCE NPs uptake reaches an equilibrium at 24 h, only slightly increasing between 24 and 48 h (Supplementary Fig. 5). It is therefore rational to postulate that enhanced cellular uptake may improve the cytotoxicity of INC280-PFCE NPs. To test this hypothesis, we added INC280-PFCE NPs and free INC280 at a gradient of INC280 concentrations into medium. Then, the viability of EBC-1 cells was analyzed by MTT assay. INC280-PFCE NPs and free INC280 exhibited cytotoxicity in a time- and concentration-dependent manner with comparable maximal therapeutic effects (Fig. 2C, Supplementary Fig. 6). The half maximal inhibitory concentration (IC_50_) values of INC280-PFCE NPs and free INC280 decreased with increasing exposure time (24 h, 48 h and 72 h) (Supplementary Table 2). Additionally, the PFCE NPs showed negligible cytotoxicity to EBC-1 cells in terms of viability even at a high concentration (1000 nM) after 72 h of incubation (Supplementary Fig. 7). These data demonstrated that INC280-PFCE NPs had notable cytotoxicity to EBC-1 cells and that PFCE NPs had good biocompatibility. In order to demonstrate the molecular mechanism of INC280-PFCE NPs to inhibit tumor growth, survival, and invasion, we analyzed the expression levels of phospho-MET and its downstream effectors using western blot. Both INC280-PFCE NPs and free INC280 resulted in the downregulation of phospho-MET as well as phospho-AKT and phospho-ERK in EBC-1 cells (Fig. 2D, Supplementary Fig. 8). Terminal deoxynucleotidyl transferase (TdT)-mediated dUTP nick-end labeling (TUNEL) assays, which can detect nuclear DNA fragmentation during apoptosis, were conducted on EBC-1 cells at different time points (Fig. 2E, Supplementary Figs. 9 and 10). In addition, apoptosis in EBC-1 cells was also assessed by flow cytometry. INC280-PFCE NPs effectively suppressed apoptosis of EBC-1 cells in a time-dependent way (Supplementary Fig. 11), and the inhibitory activity of INC280-PFCE NPs was significantly greater than that of INC280 at 72 h (Fig. 2F and G). To further study the mechanisms by which INC280-PFCE NPs cause cell death, the cell cycle analysis was conducted by flow cytometry. INC280-PFCE NPs displayed an obvious effect on the cell cycle in EBC-1 cells, which were significantly arrested in G1 phase (Fig. 2H and I, Supplementary Fig. 12). Together, these findings suggest that INC280-PFCE NPs, via inhibiting c-MET kinase activity, could efficiently block the c-MET-mediated signaling cascades. These cascades responses are essential to promote tumor growth, survival, and invasion. Moreover, INC280-PFCE NPs potently induced apoptosis in vitro, which may lead to the greatest therapeutic benefits against NSCLC progression and metastasis in vivo.

### Pulmonary delivery of INC280-PFCE NPs alters INC280 distribution behavior in orthotopic NSCLC model

**Fig. 3:**
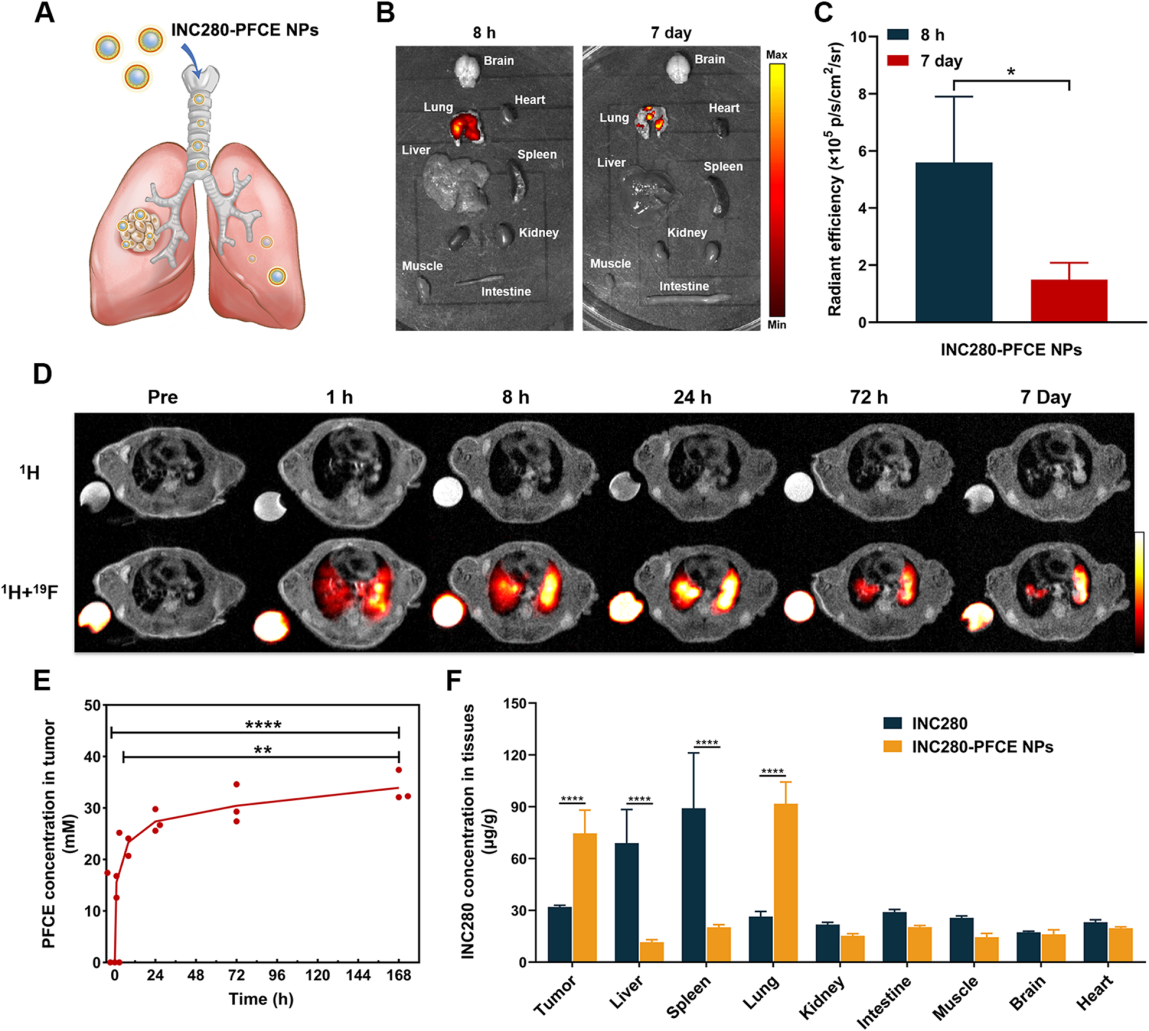
Pulmonary delivery of INC280-PFCE NPs alters INC280 distribution behavior in orthotopic NSCLC model. **A** Schematic diagram of pulmonary delivery of the INC280-PFCE NPs. **B** At 8 h and 7 days post-pulmonary delivery of INC280-PFCE NPs, ex vivo fluorescence images of tumors and major organs were taken. **C** Quantified fluorescence intensities of the excised major organs at 8 h and 7 days post-pulmonary delivery (*n* = 3). **D** In vivo ^19^F-MRI at different times after pulmonary delivery of INC280-PFCE NPs. **E** Quantitative analysis of the in vivo tumor accumulation of INC280-PFCE NPs at different times (*n* = 3). **F** After 24 h of oral administration of INC280 and pulmonary delivery of INC280-PFCE NPs, the content of INC280 in tumors and major organs was analyzed by HPLC (*n* = 3). The results are presented as mean ± standard deviation. * *P* < 0.05, ** *P* < 0.01 and **** *P* < 0.0001

To demonstrate the advantages of pulmonary delivery of INC280-PFCE NPs, we assessed their biodistribution using ex vivo fluorescence imaging. INC280-PFCE NPs labeled with rhodamine were administered by pulmonary delivery in an orthotopic NSCLC model (Fig. 3A). The fluorescent signal was predominantly clustered in the lungs at 8 h, but significantly decreased at 7 days (Fig. 3B and C). Pulmonary delivery of PFCE NPs also showed similar results (Supplementary Fig. 13). To further assess the long-term biodistribution and metabolism of INC280-PFCE NPs after IT administration, ^19^F-NMR were conducted on healthy mouse models. After pulmonary delivery of a single 50 µL dose, INC280-PFCE NPs mainly accumulate in the lung, and gradually excreted through the lungs (Supplementary Fig. 14). On the basis of their excellent ^19^F-MRI properties, we also assessed the accumulation of the INC280-PFCE NPs in the lung tumors at 1 h, 8 h, 24 h, 72 h and 7 days by ^19^F-MRI. After pulmonary delivery of INC280-PFCE NPs for 1 h, the ^19^F-MR signal was found throughout the lungs and there was a slight ^19^F-MRI signal in tumors (Fig. 3D). At 7 days, the lung tumor displayed the strongest ^19^F-MR signal and quantitative analysis of the intratumoral PFCE concentration was consistent with the ^19^F-MRI results (Fig. 3E), suggesting that the INC280-PFCE NPs were well distributed in the lung tumors and could be retained for a long time. Moreover, at 24 h posttreatment, the distribution of INC280 in the tumors and major organs was determined by high-performance liquid chromatography (HPLC). The results showed a 2.3-fold higher drug concentration of INC280-PFCE NPs group in the tumors than that of free INC280 group (Fig. 3F). Pulmonary delivery significantly reduced drug accumulation in the other organs, especially the liver and spleen. These data demonstrated that pulmonary delivery of INC280-PFCE NPs successfully altered the distribution of the small molecule inhibitor INC280 in an orthotopic NSCLC model. By ^19^F-MRI, the pulmonary delivery of INC280-PFCE NPs could be visualized and quantified in an orthotopic NSCLC model. The higher concentration of INC280 in tumors and more favorable distribution to the lungs are expected to improve efficacy in the subsequent treatment.

### Pulmonary delivery of INC280-PFCE NPs inhibits NSCLC progression in an orthotopic model

**Fig. 4:**
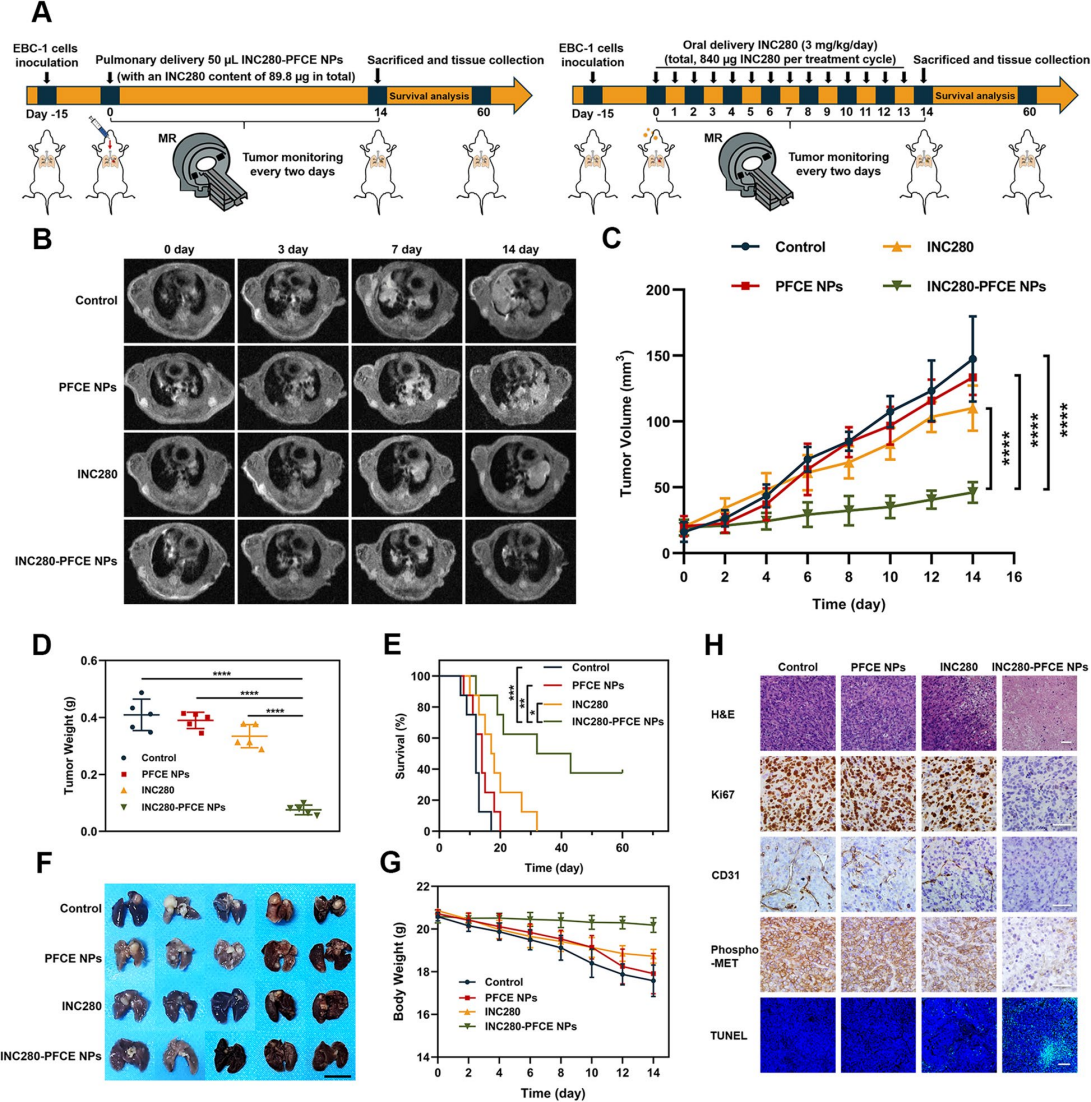
Pulmonary delivery of INC280-PFCE NPs inhibits orthotopic NSCLC progression. **A** Schematic diagram showing the treatment regimen for the model animals with orthotopic NSCLC. The mice were divided into 4 groups, Control, PFCE NPs, INC280 and INC280-PFCE NPs (n = 13 per group). PFCE NPs group: a single 50 µL of PFCE NPs was administered by IT instillation. INC280 group: free INC280 was orally given at 3 mg/kg every day and total drug doses were 840 µg in a 14-day treatment cycle. INC280-PFCE NPs group: a single dose 50 µL of INC280-PFCE NPs (with an INC280 content of 89.8 µg in total per treatment cycle of 14 days) groups were administered by IT instillation. **B** After different treatments, representative T1-weighted MR images of the mice at different time points. **C** Individual tumor growth curves of mice treated with PBS, PFCE NPs, INC280 or INC280-PFCE NPs (*n* = 5). **D** After 14 days of treatment, the weights of the tumors of the orthotopic NSCLC model mice (*n* = 5). **E** Survival curves of the tumor-bearing mice after receiving different treatments (*n* = 8). **F** On day 14 posttreatment, representative photos of lung tumors excised from the orthotopic NSCLC model mice after different treatments. The scale bar is 1 cm. **G** Body weights of the orthotopic NSCLC model mice after different treatments as a function of time (*n* = 5). **H** Histopathological analysis of the tumors harvested from the orthotopic NSCLC model mice after different treatments. Scale bar is 100 µm. The results are presented as mean ± standard deviation. * *P* < 0.05, ** *P* < 0.01, *** *P* < 0.001 and **** *P* < 0.0001

Based on the above validation that pulmonary delivery of INC280-PFCE NPs could achieve longer retention and higher drug concentration in tumors, we further examined whether a single, low therapeutic dose of INC280-PFCE NPs (50 µL of INC280-PFCE NPs with an INC280 content of 89.8 µg in total per treatment cycle) would have an objective therapeutic response in orthotopic NSCLC models. Tumor volume was monitored and quantified by ^1^H-MRI to evaluate the antitumor activities of different treatments (Fig. 4A). After a 14-day treatment, the mice administered phosphate-buffered saline (PBS; control), PFCE NPs and free INC280 (the content of INC280 was 840 µg in total per treatment cycle) exhibited rapid tumor progression. Conversely, all mice given INC280-PFCE NPs exhibited remarkable suppression of orthotopic NSCLC tumor growth (Fig. 4B and C). Additionally, INC280-PFCE NPs administration resulted in a significant decrease in tumor weight, which was more efficient than treatment with a tenfold higher dose of free INC280 (Fig. 4D) (*P* < 0.0001). As illustrated in Fig. 4E, oral administration of high-dose free INC280 every day resulted in limited survival prolongation. Nevertheless, encouragingly, a single administration of INC280-PFCE NPs significantly prolonged mouse survival compared with that in the other treatment groups, and the three mice were still alive after 60 days. At the end of the therapy period, mouse lung tumor tissues were collected. The photos in Fig. 4F further verified the superior antitumor ability of the INC280-PFCE NPs. Compared to the other three treatment groups, no significant changes in weight were observed in the mice treated with INC280-PFCE NPs, which demonstrated that the INC280-PFCE NPs caused negligible systemic toxicity (Fig. 4G). We also performed histopathological analysis on the excised tumor tissues (Fig. 4H). Hematoxylin and eosin (H&E) staining indicated that the INC280-PFCE NPs led to substantial destruction and necrosis in the lung tumors compared with other treatment groups. In addition, a significant reduction in Ki67 + tumor cell proliferation and regression of CD31 + vasculature was observed. More importantly, INC280-PFCE NPs notably reduced the expression of phospho-MET, which has been associated with poor prognosis [25]. Furthermore, from the TUNEL assays, the INC280-PFCE NPs exhibited a significant increase in green fluorescence, indicating they induced the most tumor apoptosis. Taken together, these results showed that compared to high doses of free INC280, a single pulmonary administration of INC280-PFCE NPs exhibited significantly higher drug concentration in tumors and reduced tumor growth, systematically improving the treatment effect in orthotopic NSCLC models.

### Therapeutic efficacy of image-guided INC280-PFCE NPs in NSCLC liver metastasis model

**Fig. 5:**
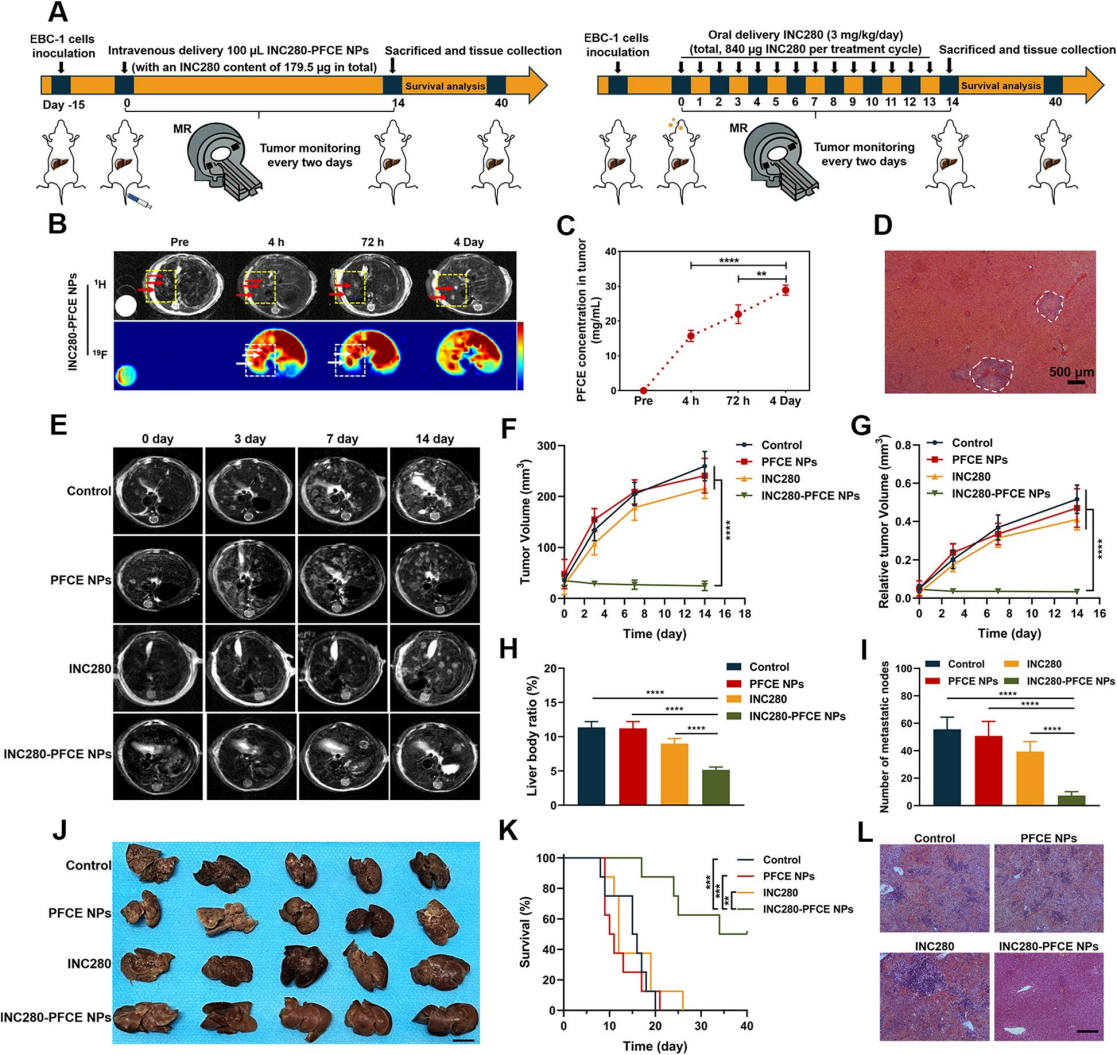
Therapeutic efficacy of image-guided INC280-PFCE NPs in NSCLC liver metastasis. **A** Schematic showing NSCLC liver metastasis model imaging and the treatment schedule. The mice were divided into 4 groups, Control, PFCE NPs, INC280 and INC280-PFCE NPs (*n* = 13 per group). PFCE NPs group: a single 100 µL of PFCE NPs was administered by IV injection. INC280 group: free INC280 was orally given at 3 mg/kg every day and total drug doses were 840 µg in a 14-day treatment cycle. INC280-PFCE NPs group: a single dose 100 µL of INC280-PFCE NPs group (with an INC280 content of 179.5 µg in total per treatment cycle of 14 days) was administered by IV injection. **B** In vivo ^19^F-MRI after IV injection of INC280-PFCE NPs. **C** Quantification of the accumulation of INC280-PFCE NPs in tumors (*n* = 3). **D** H&E staining of NSCLC liver metastasis displaying two metastatic lesions circled with white dotted lines. (scale bar = 500 µm) **E** Representative T2-weighted MRI of mice at different times. **F** Quantification of the volume of metastases from the T2-weighted MR images of mice (*n* = 5). **G** Quantification of the volumes of the livers that were occupied by metastases (*n* = 5). **H** The liver/body ratios, a parameter of tumor burden, among the four groups were compared on day 14 posttreatment (*n* = 5). **I** Quantitative analysis of the metastatic nodes number in the T2-weighted MR images from mice receiving different treatments (*n* = 5). **J** Gross appearance of NSCLC liver metastases following different therapies. The scale bar is 1 cm. **K** Survival curves of NSCLC liver metastasis model mice (*n* = 8). **L** At the end of therapy, H&E staining of the livers. Scale bar is 100 µm. The results are presented as mean ± standard deviation. **P < 0.01, ***P < 0.001, and ****P < 0.0001

We also systematically assessed the antimetastatic efficacy of INC280-PFCE NPs in NSCLC liver metastasis models, which are known to be more defensive and refractory to conventional chemotherapy than primary tumors. The schedule of imaging and therapy was displayed in Fig. 5A. When liver metastatic nodules were detected by MRI, 100 µL of INC280-PFCE NPs were injected via tail vein for imaging. INC280-PFCE NPs accumulated in the tumors at 4 h and the ^19^F-MR signal in the tumors gradually increased over time, while the ^19^F-MR signal in the livers gradually decreased (Fig. 5B). It is particularly noteworthy that the intense ^19^F-MR signal in the tumors remained stable for 4 days. Quantitative analyses of intratumoral PFCE concentration were coincide with the ^19^F-MRI results (Fig. 5C). Moreover, these metastatic lesions, detected by ^19^F-MRI with INC280-PFCE NPs, were further validated by H&E staining (labeled by the white dotted line in Fig. 5D). These data suggested that INC280-PFCE NPs can successfully reach tumors, remain there for a prolonged period of time and be readily detected by ^19^F-MRI, which provides significant guidance for subsequent treatment. To evaluate the long-term biodistribution and metabolism of INC280-PFCE NPs after IV administration, ^19^F-NMR were conducted on healthy mouse models. After IV delivery of a single 100 µL dose, INC280-PFCE NPs mainly accumulated in the liver and spleen, and the ^19^F signal was also observed in the lung, from where NPs were exhaled (Supplementary Fig. 15). On the basis of the above findings, we further validated the therapeutic efficacy of INC280-PFCE NPs following a single low-dose IV injection (100 µL of INC280-PFCE NPs with an INC280 content of 179.5 µg in total per treatment cycle) in NSCLC liver metastasis models. ^1^H-MRI confirmed that metastatic progress was quick with metastatic lesions scattered throughout the liver (Fig. 5E). The entire liver seemed to be fully invaded and exhibited diffuse changes in the PBS (control), PFCE NPs and high-dose free INC280 (the content of INC280 was 840.0 µg in total per treatment cycle) groups after 14 days. However, INC280-PFCE NPs significantly suppressed metastatic progression. Moreover, INC280-PFCE NPs exhibited robust tumor inhibition compared to orally administered high-dose free INC280 (Fig. 5F and G). Compared with the other three groups, treatment with INC280-PFCE NPs significantly lowered the liver/body ratio (Fig. 5H, *P* < 0.0001). The liver weights of healthy nude mice ranged between 0.9 and 1.2 g, which was similar to that in the mice treated with INC280-PFCE NPs. In addition, INC280-PFCE NPs significantly reduced the number of metastatic nodes (Fig. 5I, *P* < 0.0001). The gross appearance of NSCLC liver metastasis following different treatments correlated with the imaging data (Fig. 5J). The above indicators are all favorable for improving prognosis and prolonging survival (Fig. 5K). Then, the livers were excised for histopathological analysis. PBS, PFCE NPs and free INC280 led to increased metastases and the destruction of the entire liver tissue (H&E staining) (Fig. 5L). However, INC280-PFCE NPs resulted in fewer metastases and slight liver tissue destruction. Furthermore, the Ki67 immunohistochemical (IHC) assays of the liver tissues revealed that INC280-PFCE NPs inhibited the growth of metastases more efficiently than free INC280 (Supplementary Fig. 16). Additionally, the prolonged retention of NPs in the tumors led to improved tumor growth control in NSCLC liver metastasis models compared with the traditional formulation of INC280. These findings indicate that a single low dose of INC280-PFCE NPs administered intravenously leads to significant and sustained in vivo inhibitory activity against NSCLC liver metastasis and provides an alternative to oral treatment with INC280 for advanced NSCLC.

### Clinical translational application of INC280-PFCE NPs

**Fig. 6:**
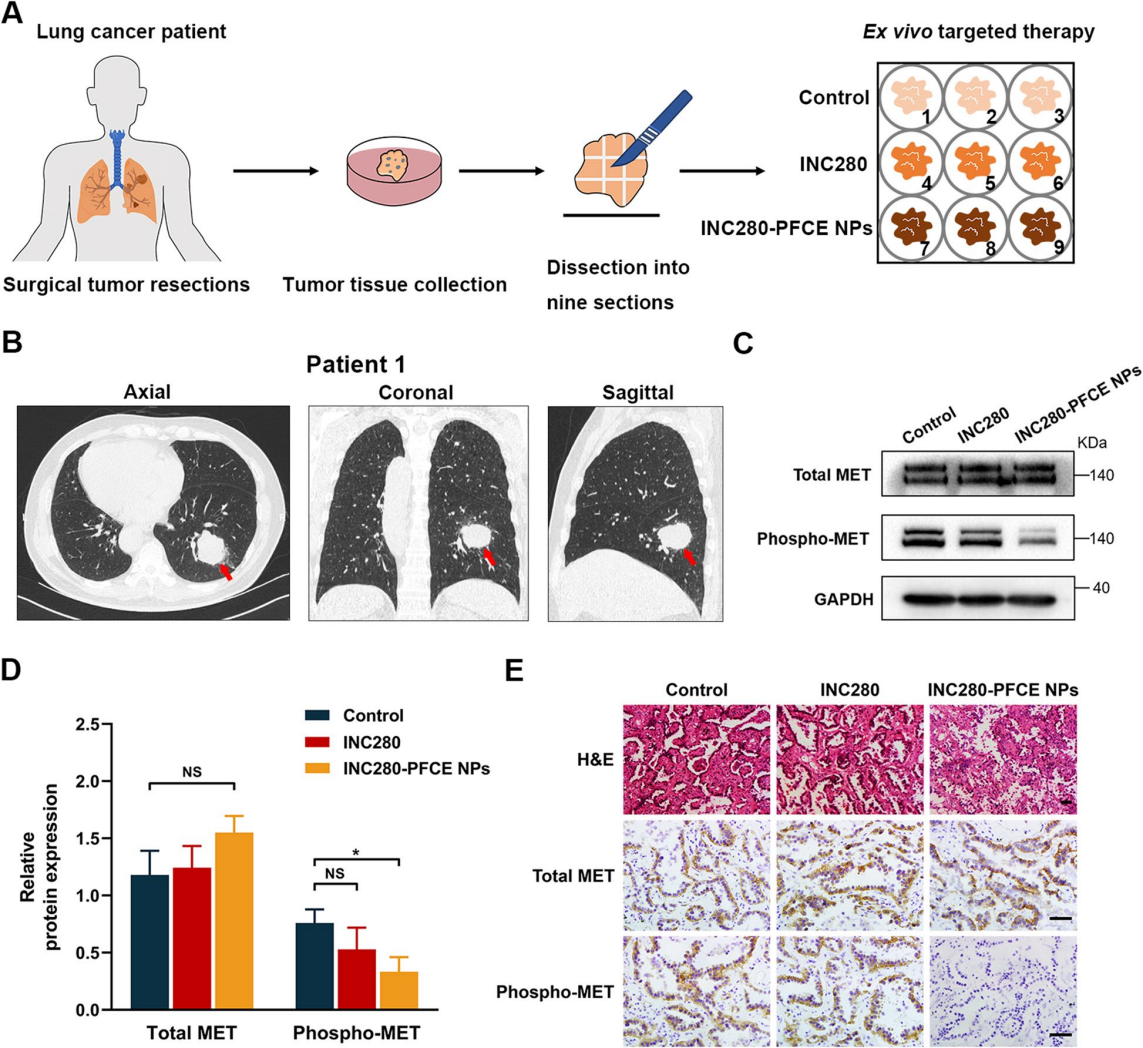
Clinical translational potential of INC280-PFCE NPs. **A** Ex vivo experiment with PDTFs. Freshly surgically resected lung cancer tissues were divided into 9 fragments, each of which was 40 ~ 50 mm^3^ in size, and randomly cultured in medium with PBS, INC280 or INC280-PFCE NPs for 48 h. The groups of INC280 and INC280-PFCE NPs had the same concentration of INC280 (10 nM). **B** Representative computed tomography (CT) images of a 68-year-old aging patient diagnosed with NSCLC (red arrow, tumor). **C-D** After the PDTFs were processed for 48 h, the expression of total MET and phospho-MET was verified and quantified by western blot. GAPDH was selected as the internal control. **E** Histopathological analysis (H&E staining and IHC assay) of the PDTFs after 48 h of ex vivo targeted therapy. Scale bar = 100 µm. Data are presented as mean ± standard deviation (n = 6). * *P* < 0.05

Given the promising preclinical results, we further conducted clinically relevant validation studies using patient-derived tumor fragments (PDTFs) for NSCLC treatment to demonstrate the clinical translational feasibility of INC280-PFCE NPs. We tested 6 independent human NSCLC specimens that expressed MET (Supplementary Table 3). Human lung cancer specimens were acquired from patients undergoing surgery and dissected into fragments. To reduce the confounding effects of tumor heterogeneity, the PDTFs were randomly cultured ex vivo in medium with PBS, INC280 or INC280-PFCE NPs for 48 h (Fig. 6A). The first tumor sample was from a 68-year-old aging male with a lesion located in the lower lobe of the right lung (Fig. 6B). After 48 h of ex vivo treatment, we verified the expression levels of total MET and phospho-MET. As illustrated in Fig. 6C and D, INC280-PFCE NPs significantly lowered the expression of phospho-MET (*P* < 0.05). Additionally, histopathological analyses suggested that substantial destruction of lung tumors were observed and phospho-MET expression were significantly reduced in the PDTFs treated with INC280-PFCE NPs, which was consistent with the results of western blot (Fig. 6E). The same therapeutic effects were observed in tumor samples from 5 other NSCLC patients (Supplementary Fig. 17A-E). As mentioned above, we used novel PDTFs to demonstrate that INC280-PFCE NPs could improve the therapy efficacy against human lung cancer, which has great potential for clinical translation.

### Safety evaluation

First, the in vivo toxicities of INC280-PFCE NPs or PFCE NPs administered by pulmonary and IV delivery were determined in healthy BALB/c nude mice. The mice administered a therapeutic dose of INC280-PFCE NPs or PFCE NPs demonstrated no obvious hematological toxicity on days 1, 7 and 30 posttreatment (Supplementary Figs. 18 and 19). Since pulmonary delivery of INC280-PFCE NPs previously showed significantly higher INC280 content in the lungs than orally administered free INC280, we studied the effect of pulmonary delivery of INC280-PFCE NPs on lung injury on days 1, 7 and 30 posttreatment. As displayed in Supplementary Fig. 20 and Supplementary Fig. 21, pulmonary administration of INC280-PFCE NPs and PFCE NPs did not cause notable pathological changes or pulmonary fibrosis and collagen fiber deposition as determined by H&E staining and Masson trichrome staining, respectively. Additionally, no significant differences of cytokines in the lungs were observed on days 1, 7 or 30 posttreatment (Supplementary Fig. 22). These data demonstrate that the therapeutic dose of INC280-PFCE NPs is feasible and safe and causes no damage to the lungs. Then, we performed toxicity studies on orthotopic NSCLC and NSCLC liver metastasis models after a 14-day treatment regimen. The mice orally administered high doses of free INC280 every day showed increased serum aspartate aminotransferase (AST), alanine aminotransferase (ALT), blood urea nitrogen (BUN) and creatinine (Crea) levels (Supplementary Figs. 23 and 24). Conversely, a single low dose of INC280-PFCE NPs demonstrated no significant hepatotoxicity or nephrotoxicity. Furthermore, H&E staining indicated that the different treatments caused no apparent organ damage in the orthotopic NSCLC models (Supplementary Fig. 25). In summary, the safety profile of INC280-PFCE NPs compared with that of free INC280 indicates a favorable shift in the toxicity profile for the NP-encapsulated drug.

## Discussion

Currently, MET is a key target in therapeutic blockade of NSCLC [26]. On Aug 10, 2022, INC280 obtained the FDA regular approval for the treatment of advanced NSCLC[8]. Despite the promising efficacy data for INC280, its clinical utility after oral administration is hampered by poor target specificity and bioavailability and systemic toxic effects. In addition, 67% of patients suffer adverse events when in grade 3 or 4 [10]. The reason for these results is the limited capability of the drugs to reach the tumor cells at a lethal concentration [27]. Due to its extreme water insolubility, INC280 cannot be delivered by intratracheal (IT) or IV routes and therefore must be given by an oral dosing regimen. Cancer treatments given orally are usually restricted by 1) inability to circumvent biological barriers, especially in the liver and spleen, 2) a low degree of drug accumulation in lung tumors, 3) ineffectiveness against metastatic disease, and 4) a lack of long-term effective treatment monitoring [28–31]. For INC280, even repeated daily oral administration fails to increase drug concentrations in primary and metastatic lesions. Additionally, the resulting cumulative amount of anticancer drug may produce systemic toxicity. Therefore, there is an urgent need for therapeutic agents with the dual functions of targeted therapy and in vivo molecular imaging capability, which could identify tumor cell location and aid in improving treatment regimens [32].

PFCE NPs, as superior nanoplatforms, are often modified with active targeting ligands or antibodies for targeted diagnosis [33–35]. However, there are few studies on their encapsulation of small-molecule inhibitors due to the extreme hydrophobicity of these drugs. In this study, we studied the use of a PFCE NP-based drug delivery vectors to upgrade the solubility and biocompatibility of the small molecule inhibitor INC280 to enhance its anti-MET/antimetastatic activities. Specifically, aiming to optimize the hydrophobicity of INC280 and maximize the fluorine content in the INC280-PFCE NPs, the PFCE NPs were stabilized in vivo with natural phospholipids. This strategy of synthesis greatly limits the PFCE cores to coalesce with each other. These natural phospholipids offer an optimal hydrophilic-lipophilic balance and lower the PFCE/water interfacial tension [36]. Moreover, the phospholipid layer provides an appropriate location and specific surface area for maximum INC280 loading and quite different from modification with targeting ligands attached by chemical reactions between functional groups [37]. On the one hand, loading drugs in this way will not influence the chemical structure of the small molecule inhibitor INC280 and its interaction with MET, allowing effective targeted therapy by interacting with invasion-related proteins. We have confirmed that the excellent on-target antitumor activity of the INC280-PFCE NPs was not compromised in both in vivo and in vitro studies. On the other hand, because the biocompatibility and stability of the particles were improved, the INC280-PFCE NPs altered the distribution of INC280 in vivo. Studies have confirmed that, after oral dosing, INC280 was systemically available, largely distributed in the peripheral tissues and ultimately eliminated through metabolism and biliary/fecal and renal excretion [38]. Our findings demonstrated that pulmonary delivery of INC280-PFCE NPs primarily targeted the drug distribution to the lungs and tumors. Additionally, the INC280-PFCE NPs showed a twofold higher intratumor drug concentration than that after oral administration of free INC280. Moreover, compared to the rapid blood clearance of the small molecule inhibitor INC280 in its free form, a single IV dose of INC280-PFCE NPs achieved long-term retention in metastatic lesions. To further scale up the production of INC280-PFCE NPs, the synthesis was carried out by microfluidization [39]. Different from probe sonication, microfluidization is characterized by high shear, cavitation and impact, allowing the formation of particles with high monodispersity and stability [40]. The simple synthesis method, single low-dose dosing regimen, safe and nontoxic components and scaled-up production satisfy the requirements for translational applications into large animal and primate models.

Since the PFCE NPs demonstrated a satisfactory INC280 loading capacity and high biocompatibility, we further confirmed that a single low dose of INC280-PFCE NPs could achieve efficient anti-MET/antimetastatic effects via a multirooted delivery strategy, which may change the conventional administration of INC280. In orthotopic NSCLC models, a single pulmonary administration of INC280-PFCE NPs significantly inhibited tumor progression and prolonged animal survival. Moreover, in NSCLC liver metastasis models, compared with high oral dosages of free INC280, a single low dose of INC280-PFCE NPs effectively prolonged mice survival. Mechanistically, INC280-PFCE NPs effectively inhibited tumor survival, proliferation and migration by inhibiting the c-MET signaling pathway and its major downstream factors. In addition to the effective anti-MET/antimetastatic effects, a single low dose of INC280-PFCE NPs reduced not only the frequency of dosing and total doses but also the off-target toxicity, revealing its high efficiency and biosafety in vivo. More importantly, as a clinical proof of concept, we used novel PDTFs to verify that INC280-PFCE NPs can improve the therapeutic effect of INC280, demonstrating the excellent translational potential of INC280-PFCE NPs. Possible gender effects are a limitation for the clinical translation of INC280-PFCE as a drug delivery system. We will definitely explore this question in our future research.

^19^F-MRI of INC280-PFCE NPs is of great interest for tumor localization and targeted therapy, enabling real-time visualization of drug release and biodistribution in vivo. In this study, in an orthotopic NSCLC model, the rapid entrance of INC280-PFCE NPs into the lung following a single administration and homing to the cell clusters was observed by ^19^F-MRI. The fluorine signal from the INC280-PFCE NPs in the lungs was gradually metabolized and remained only in the tumors, indicating that the loaded small molecule inhibitor INC280 was also distributed in this way. We have therefore demonstrated that INC280 was stably and efficiently loaded into PFCE NPs. After IV administration, leaky capillary endothelium-associated tumor vasculature allows leakage of NPs into tumor sites through the enhanced permeability and retention effect, especially when the particle sizes are between 10 ~ 200 nm, which is more favorable to the antitumor gap pressure of the NPs [41]. In the case of liver metastases, the INC280-PFCE NPs approximately 100 nm in size gradually migrated into the tumor regions and remained at a steady level in the liver lesions for 4 days after a single IV administration, which was also readily detected by ^19^F-MRI. This further showed that the INC280-PFCE NPs displayed sustained release and prolonged retention behaviors. These dual-use INC280-PFCE NPs for both drug delivery and ^19^F-MRI in vivo may provide imaging of disease states and conclusive evidence of drug distribution to regions of interest [12]. The ability to quantify the concentration of these NPs by ^19^F-MRI may be of great benefit in estimating local drug concentration. At the same time, new pharmacokinetic and pharmacodynamic paradigms are developed to characterize such new class of drugs.

## Conclusion

In conclusion, we successfully developed a multirooted delivery system based on PFCE NPs to enhance the anti-MET/antimetastatic efficacy of INC280. In orthotopic NSCLC models, pulmonary delivery of INC280-PFCE NPs achieves sustained drug release, prolonged drug retention in tumors and real-time visualization by ^19^F-MRI. Moreover, a single pulmonary administration of INC280-PFCE NPs more efficiently inhibited tumor growth than daily treatment with high doses of free INC280. Meanwhile, the long-term retention of INC280-PFCE NPs in liver metastases could efficiently translate into inhibition of widespread dissemination after a single low-dose IV administration. On the basis of the significant reduction in the total doses, a single dose of INC280-PFCE NPs notably improved the toxicity related to high oral doses of free INC280. Finally, we validated the clinical translational potential of INC280-PFCE NPs with PDTFs. According to our knowledge, this is the first study of a multirooted drug delivery system based on PFCE NPs to improve the therapeutic efficacy of small molecule MET inhibitors in clinical applications. Consequently, we suggest that this novel strategy holds the potential to be broadly applicable to the treatment of advanced NSCLC by altering different targeted therapeutic effectors of tumors.

## Supplementary Information

**Additional file 1:** **Supplementary Figure 1. **Average hydrodynamic size of PFCE NPs characterized by dynamic light scattering (DLS). **Supplementary Figure 2. **Transmission electron microscopy (TEM) image of INC280-PFCE NPs. **Supplementary Figure 3. **The size stability test of INC280-PFCE NPs at FBS and culture medium for up to 14 days.** Supplementary Figure 4. **Quantitative analysis of the ^19^F-MR signal-to-noise ratio (SNR) versus ^19^F concentration. **Supplementary Figure 5. **PFCE NPs uptake efficiency *in vitro*, (A, B). **Supplementary Figure 6. **Viability of EBC-1 cells treated with various concentrations of INC280 for 24, 48 and 72h. **Supplementary Figure 7. **Viability of EBC-1 cells treated with various concentrations of PFCE NPs for 24, 48 and 72 h. **Supplementary Figure 8.** Quantitative analysis of the western blot results. **Supplementary Figure 9. **Quantification of TUNEL-positive cells after 72 h of treatment. **Supplementary Figure 10.** Detection of EBC-1 cell apoptosis by TUNEL assays, (a, b).**SupplementaryFigure 11. **Detection of EBC-1 cell apoptosis by flow cytometry, (a, b, c, d).**Supplementary Figure 12. **Detection of EBC-1 cell cycle distribution by flow cytometry, (a, b, c, d).**Supplementary Figure 13. **After pulmonary delivery of PFCE NPs, *ex vivo* fluorescence images of the major organs were obtained at 8 h and 7 days. **Supplementary Figure 14. **After different time periods of IT administration with INC280-PFCE NPs, ^19^F-NMR was used to measure PFCE concentration in heart, liver, spleen, lung, kidney, intestine and feces of healthy BALB/c nude mice, (A, B, C, D, E, F, G, H). **Supplementary Figure 15. **After different time periods of IV administration with INC280-PFCE NPs, ^19^F-NMR was used to measure PFCE concentration in heart, liver, spleen, lung, kidney, intestine and feces of healthy BALB/c nude mice, (A, B, C, D, E, F, G, H). **Supplementary Figure 16. **Ki67 staining of livers harvested from NSCLC liver metastasis model mice after different treatment. **Supplementary Figure 17. **After the PDTFs were processed for 48 h, the expression of total MET and phospho-MET was verified and quantified by western blot, (A, B, C, D, E). **Supplementary Figure 18. **After pulmonary delivery of PFCE NPs or INC280-PFCE NPs, blood biochemistry and hematological parameters of healthy BALB/c nude mice were measured.** Supplementary Figure 19. **After intravenous (IV) delivery of PFCE NPs or INC280-PFCE NPs, blood biochemistry and hematological parameters of healthy BALB/c nude mice were measured. **Supplementary Figure 20. **Effects of pulmonary delivery of PFCE NPs or INC280-PFCE NPs on lung damage validated by H&E staining. **Supplementary Figure 21. **Effects of pulmonary delivery of PFCE NPs orINC280-PFCE NPs on lung damage validated by Masson trichrome staining. **Supplementary Figure 22.** Effects of pulmonary delivery of PFCE NPs or INC280-PFCE NPs on lung damage validated by ELISA,(a, b, c, d). **Supplementary Figure 23. **After 14 days of treatment, (a-b) hepatotoxicity, (c-d) nephrotoxicity, and (e-f) hematological parameters from orthotopic NSCLC model mice treated with different formulations. **Supplementary Figure 24. **After 14 days of treatment, (a-b) hepatotoxicity, (c-d) nephrotoxicity, and (e-f) hematological parameters from NSCLC liver metastasis model mice treated with different formulations.** Supplementary Figure 25. **After 14 days of treatment, H&E staining was performed on the major organs of orthotopic NSCLC model mice.** Supplementary Table 1. **The particle size of INC280-PFCE NPs. **Supplementary Table 2. **The IC_50_ values of free INC280 and INC280-PFCE NPs. **Supplementary Table 3. **Patient characteristics and pathological information of all the resected samples.

**Additional file 2:** Supplementary materials and methods.

## Data Availability

All data generated or analysed during this study are included in this published article and its supplementary information files.

## Abbreviations

NSCLC: Non-small cell lung cancer
MET: Mesenchymal-epithelial transition factor
PFCE NPs: Perfluoro-15-crown-5-ether nanoparticles
FDA: Food and Drug Administration
INC280-PFCE NPs: Loading of INC280 into the PFCE NPs
^19^F-MRI: ^19^F magnetic resonance imaging
PDTFs: Patient-derived tumor fragments
IT: Intratracheal
IV: Intravenous
DLS: Dynamic Light Scattering
TEM: Transmission electron microscope
