# Supplementary material for: Single low-dose INC280-loaded theranostic nanoparticles achieve multirooted delivery for MET-targeted primary and liver metastatic NSCLC
Source: Mol Cancer. 2022 Dec 1;21:212. doi: 10.1186/s12943-022-01681-y (PMC9717478; doi:10.1186/s12943-022-01681-y)
Supplement: Supplementary file 1 — Additional file 1: Supplementary Figure 1. Average hydrodynamic size of PFCE NPs characterized by dynamic light scattering (DLS). Supplementary Figure 2. Transmission electron microscopy (TEM) image of INC280-PFCE NPs. Supplementary Figure 3. The size stability test of INC280-PFCE NPs at FBS and culture medium for up to 14 days. Supplementary Figure 4. Quantitative analysis of the 19F-MR signal-to-noise ratio (SNR) versus 19F concentration. Supplementary Figure 5. PFCE NPs uptake efficiency in vitro, (A, B). Supplementary Figure 6. Viability of EBC-1 cells treated with various concentrations of INC280 for 24, 48 and 72h. Supplementary Figure 7. Viability of EBC-1 cells treated with various concentrations of PFCE NPs for 24, 48 and 72 h. Supplementary Figure 8. Quantitative analysis of the western blot results. Supplementary Figure 9. Quantification of TUNEL-positive cells after 72 h of treatment. Supplementary Figure 10. Detection of EBC-1 cell apoptosis by TUNEL assays, (a, b).SupplementaryFigure 11. Detection of EBC-1 cell apoptosis by flow cytometry, (a, b, c, d).Supplementary Figure 12. Detection of EBC-1 cell cycle distribution by flow cytometry, (a, b, c, d).Supplementary Figure 13. After pulmonary delivery of PFCE NPs, ex vivo fluorescence images of the major organs were obtained at 8 h and 7 days. Supplementary Figure 14. After different time periods of IT administration with INC280-PFCE NPs, 19F-NMR was used to measure PFCE concentration in heart, liver, spleen, lung, kidney, intestine and feces of healthy BALB/c nude mice, (A, B, C, D, E, F, G, H). Supplementary Figure 15. After different time periods of IV administration with INC280-PFCE NPs, 19F-NMR was used to measure PFCE concentration in heart, liver, spleen, lung, kidney, intestine and feces of healthy BALB/c nude mice, (A, B, C, D, E, F, G, H). Supplementary Figure 16. Ki67 staining of livers harvested from NSCLC liver metastasis model mice after different treatment. Supplementary Figure 17. After t [file 12943_2022_1681_MOESM1_ESM.docx]

*Supplementary data*

**Single low-dose INC280-loaded theranostic nanoparticles achieve multirooted delivery for MET-targeted primary and**

**liver metastatic NSCLC**

The supplementary file includes 25 Figures and 3 Tables.

Yige Sun^1,2†^, Jie Yang^1,2†^, Yingbo Li^1,2†^, Jing Luo^1,2^, Jiemei Sun^1,2^, Daoshuang Li^1,2^, Yuchen Wang^1,2^, Kai Wang^1,2^, Lili Yang^1,2^, Lina Wu^1,2^* and Xilin Sun^1,2^*

* Correspondence: sunxl@ems.hrbmu.edu.cn; LinaWu@hrbmu.edu.cn.

^1^ Department of Nuclear Medicine, the Fourth Hospital of Harbin Medical University, 150028 Harbin, Heilongjiang, China.

^2^ NHC Key Laboratory of Molecular Probe and Targeted Diagnosis and Therapy, Molecular Imaging Research Center (MIRC), Harbin Medical University, 150028 Harbin, Heilongjiang, China.


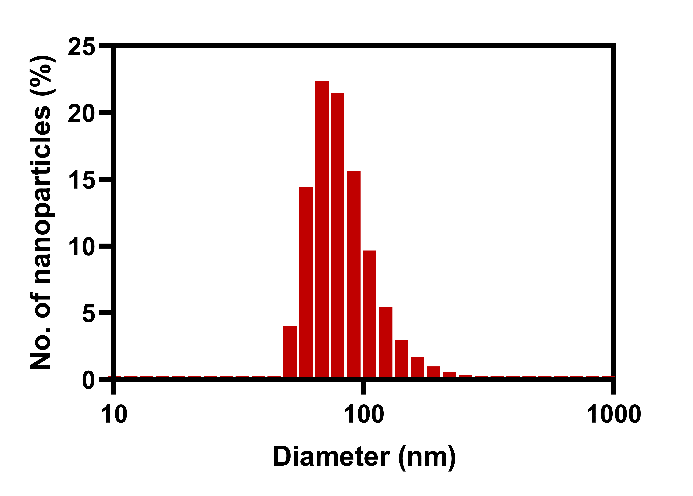


**Supplementary Fig. 1** Average hydrodynamic size of PFCE NPs characterized by dynamic light scattering (DLS).


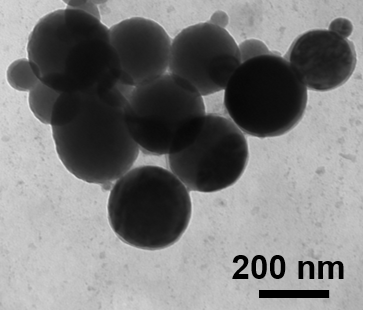


**Supplementary Fig. 2** Transmission electron microscopy (TEM) image of INC280-PFCE NPs. Scale bar = 200 nm.


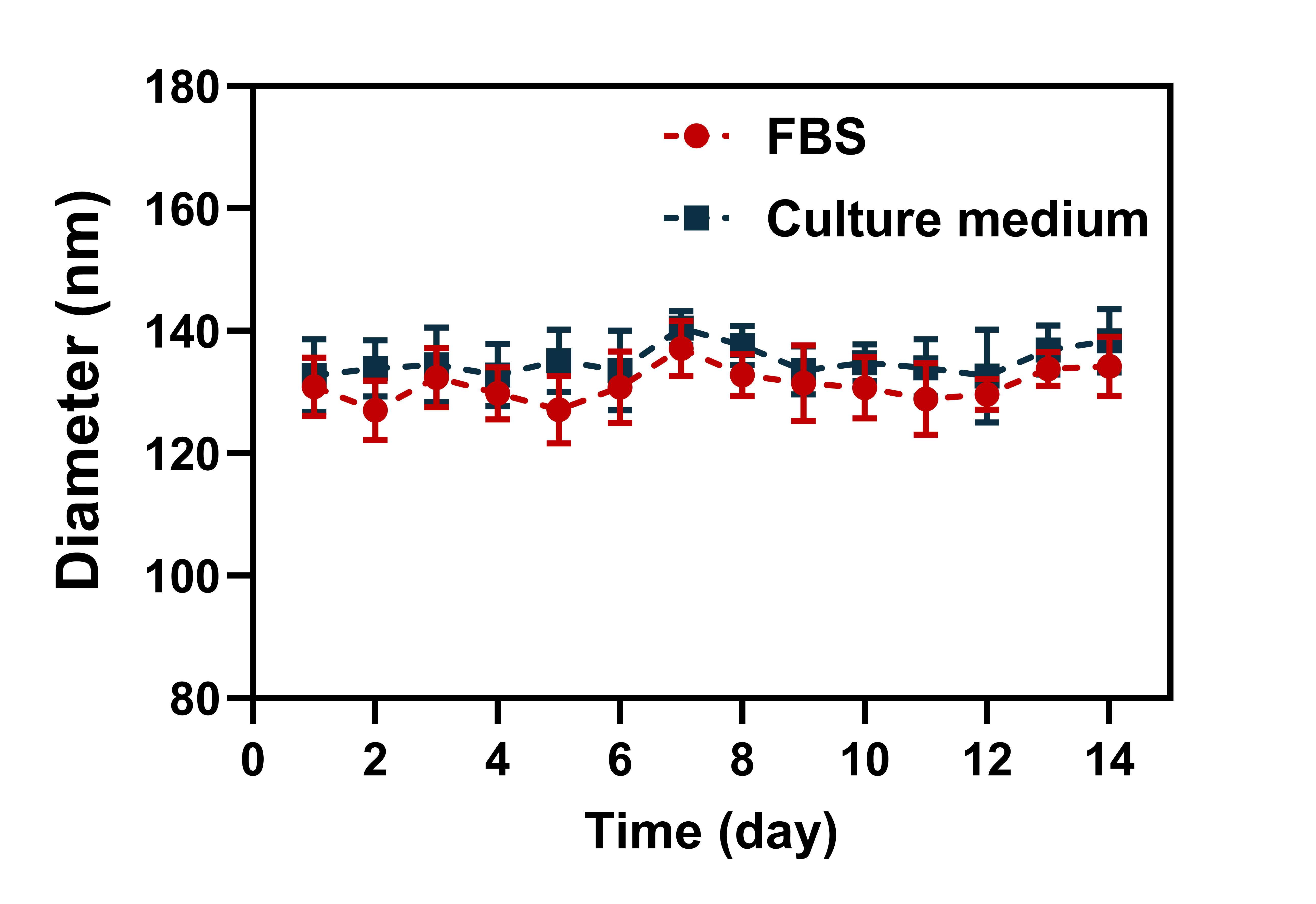


**Supplementary Fig. 3** The size stability test of INC280-PFCE NPs at FBS and culture medium for up to 14 day. Data are shown as mean ± standard deviation (n = 3).


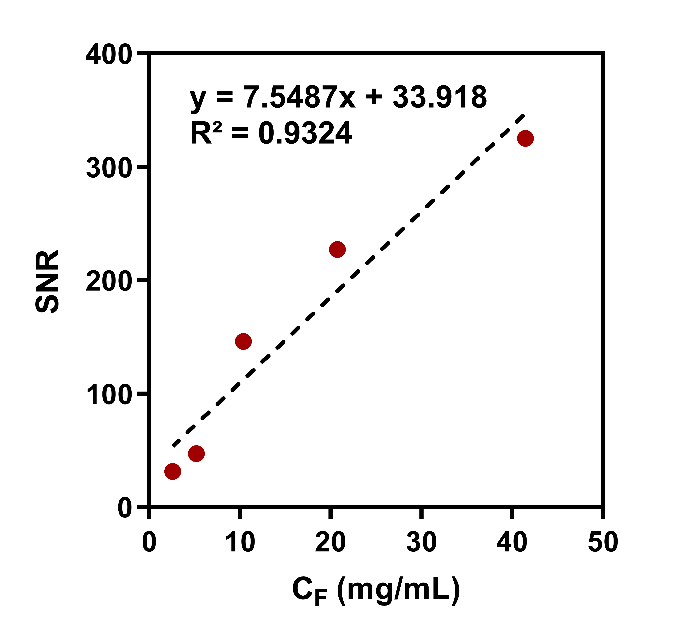


**Supplementary Fig. 4** Quantitative analysis of the ^19^F-MR signal-to-noise ratio (SNR) versus ^19^F concentration (C_F_). 1-5: 2.59, 5.19, 10.37, 20.74, 41.48 mg/mL.

**
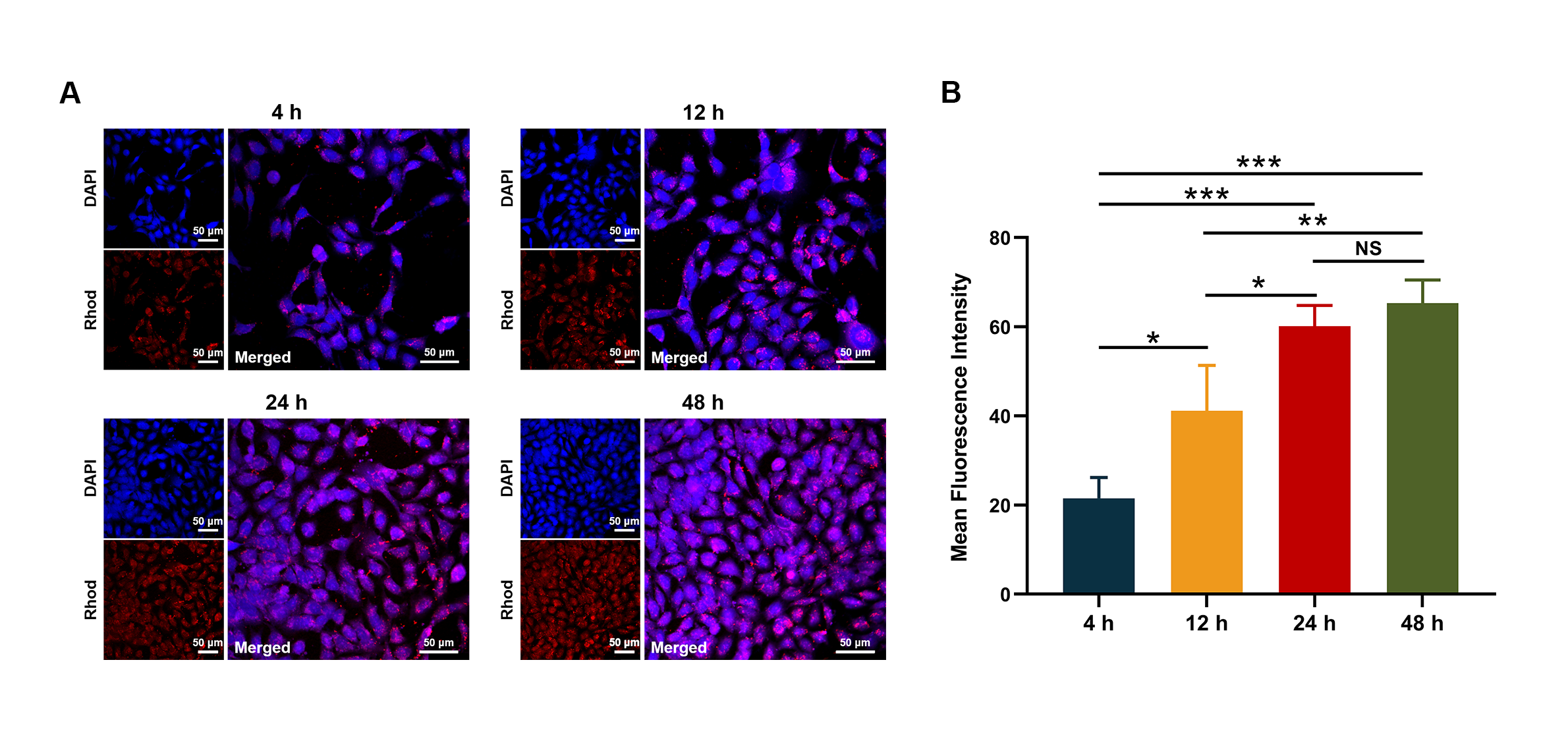
** **Supplementary Fig. 5** PFCE NPs uptake efficiency *in vitro*. **(A)** Representative CLSM images. EBC-1 cells were incubated with rhodamine-labeled PFCE NPs (red) (300 nM PFCE) for 4 h, 12 h, 24 h and 48 h. The nuclei were stained with DAPI (blue). Scale bar is 50 µm. **(B)** After incubation for 4 h, 12 h, 24 h and 48 h, analysis of mean fluorescence intensity (MFI) of PFCE NPs in EBC-1 cells. Data are presented as mean ± standard deviation (n = 3). * *P* < 0.05, ** *P* < 0.01 and *** *P* < 0.001.


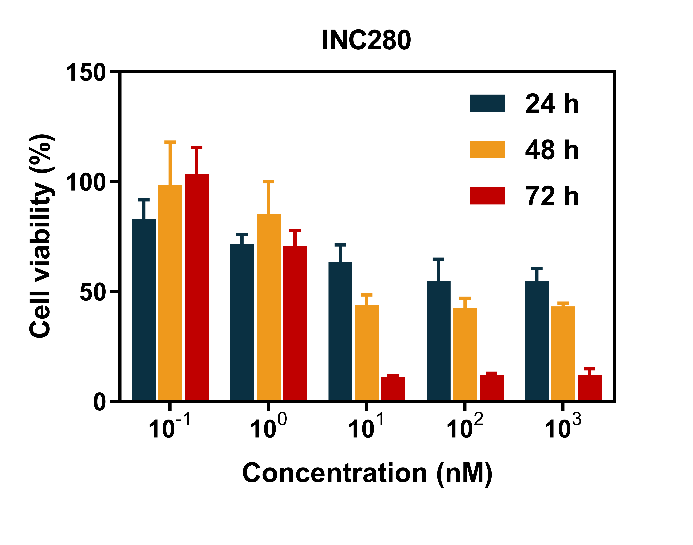


**Supplementary Fig. 6** Viability of EBC-1 cells treated with various concentrations of INC280 for 24, 48 and 72 h.


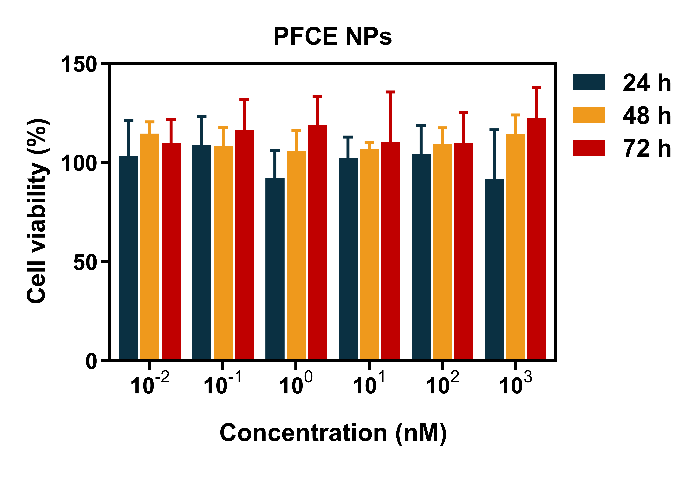


**Supplementary Fig. 7** Viability of EBC-1 cells treated with various concentrations of PFCE NPs for 24, 48 and 72 h.


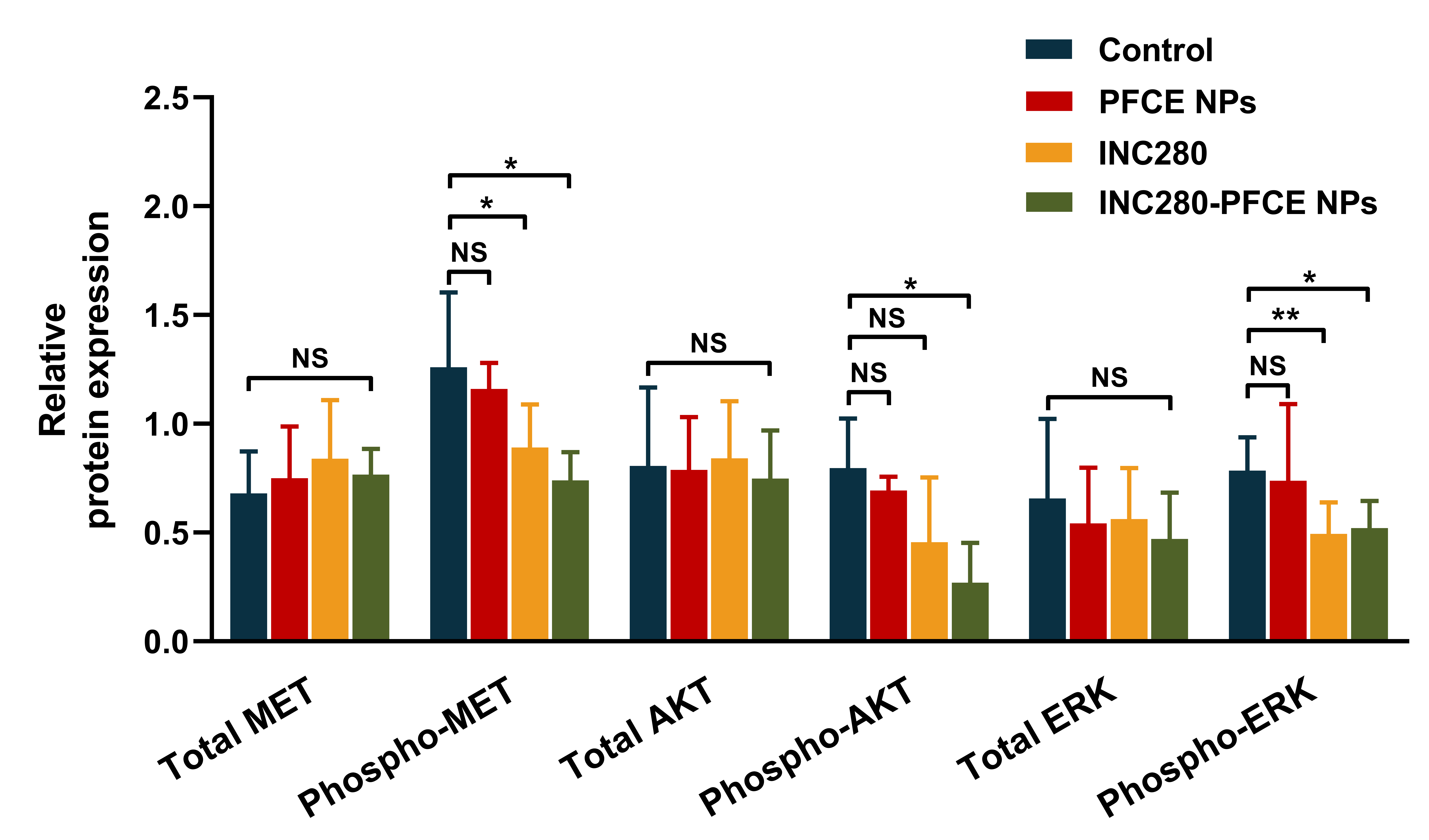


**Supplementary Fig. 8** Quantitative analysis of the western blot results. GAPDH was used as the loading control for each sample. Data are presented as mean ± standard deviation (n = 3). * *P* < 0.05 and ** *P* < 0.01.


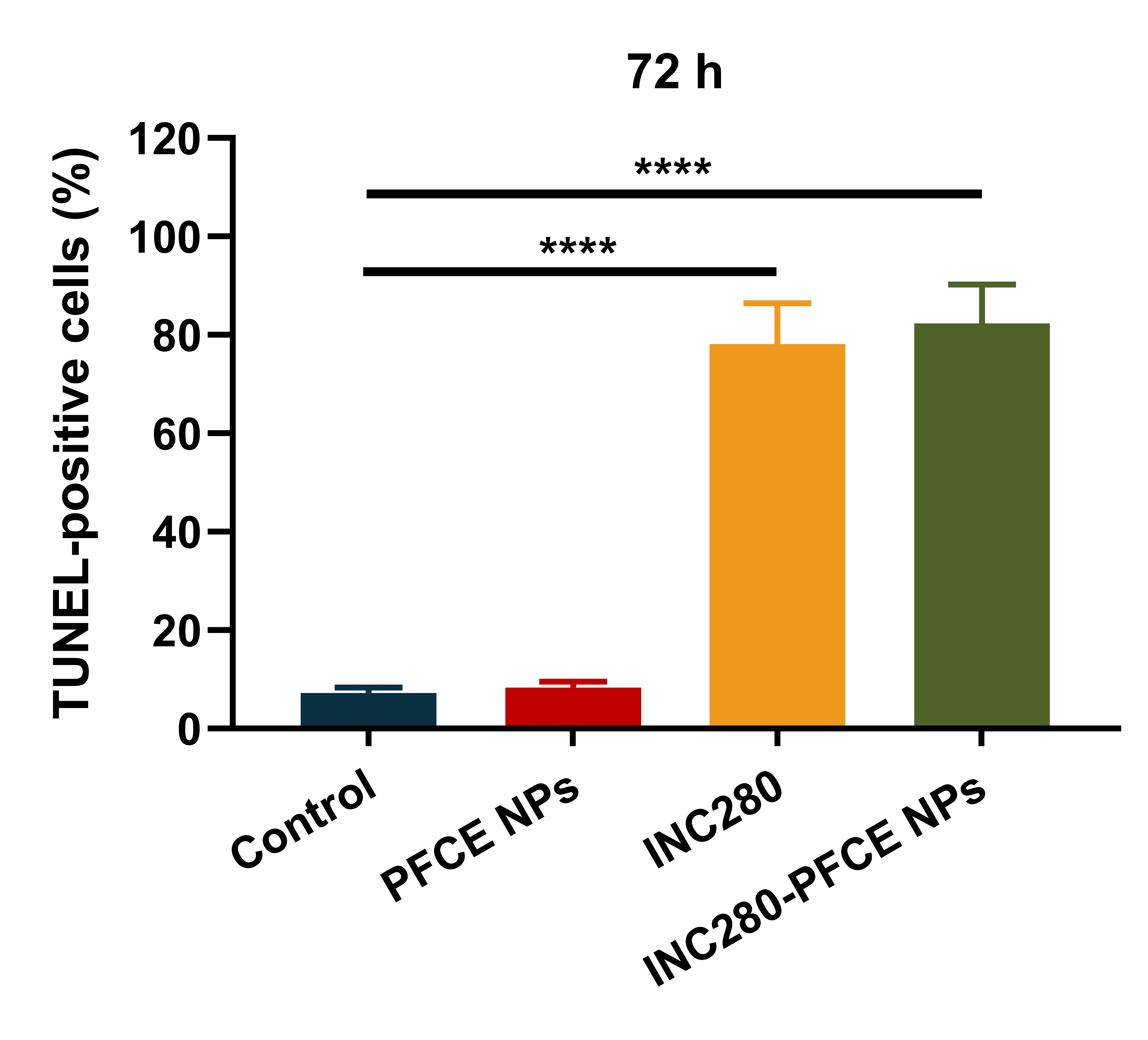


**Supplementary Fig. 9** Quantification of TUNEL-positive cells after 72 h of treatment. Control, PFCE NPs (300 nM PFCE), INC280 (10 nM INC280) and INC280-PFCE NPs (10 nM INC280 and 300 nM PFCE). Data are shown as mean ± standard deviation (n = 3; **** *P* < 0.0001).


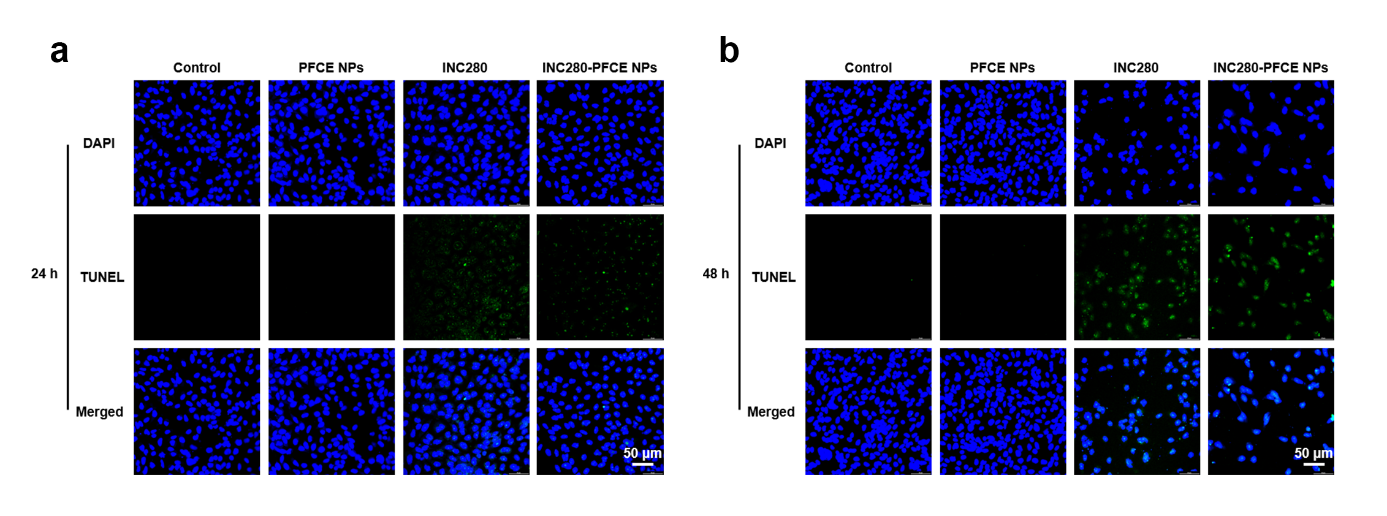


**Supplementary Fig. 10** Detection of EBC-1 cell apoptosis by TUNEL assays. (a) Detection of EBC-1 cell apoptosis by TUNEL assays after treatment with different formulations for 24 h. Scale bar is 50 µm. (b) Detection of EBC-1 cell apoptosis by TUNEL assays after treatment with different formulations for 48 h. Scale bar is 50 µm.


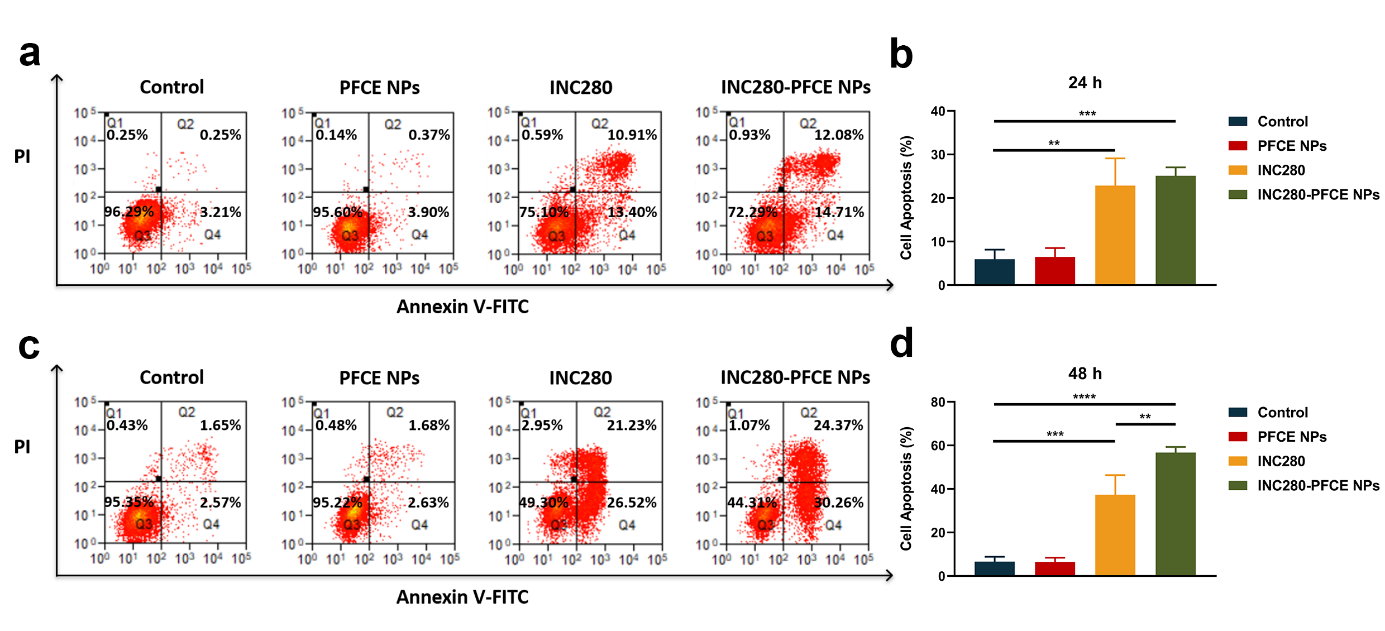


**Supplementary Fig. 11** Detection of EBC-1 cell apoptosis by flow cytometry. (a) Detection of EBC-1 cell apoptosis by flow cytometry after treatment with different formulations for 24 h. (b) Quantification of cell apoptosis measured by flow cytometry after 24 h. (c) Detection of EBC-1 cell apoptosis by flow cytometry after treatment with different formulations for 48 h. (d) Quantification of cell apoptosis measured by flow cytometry after 48 h. Data are shown as the mean ± standard deviation (n = 3; ** *P* < 0.01, *** *P* < 0.001 and **** *P* < 0.0001).


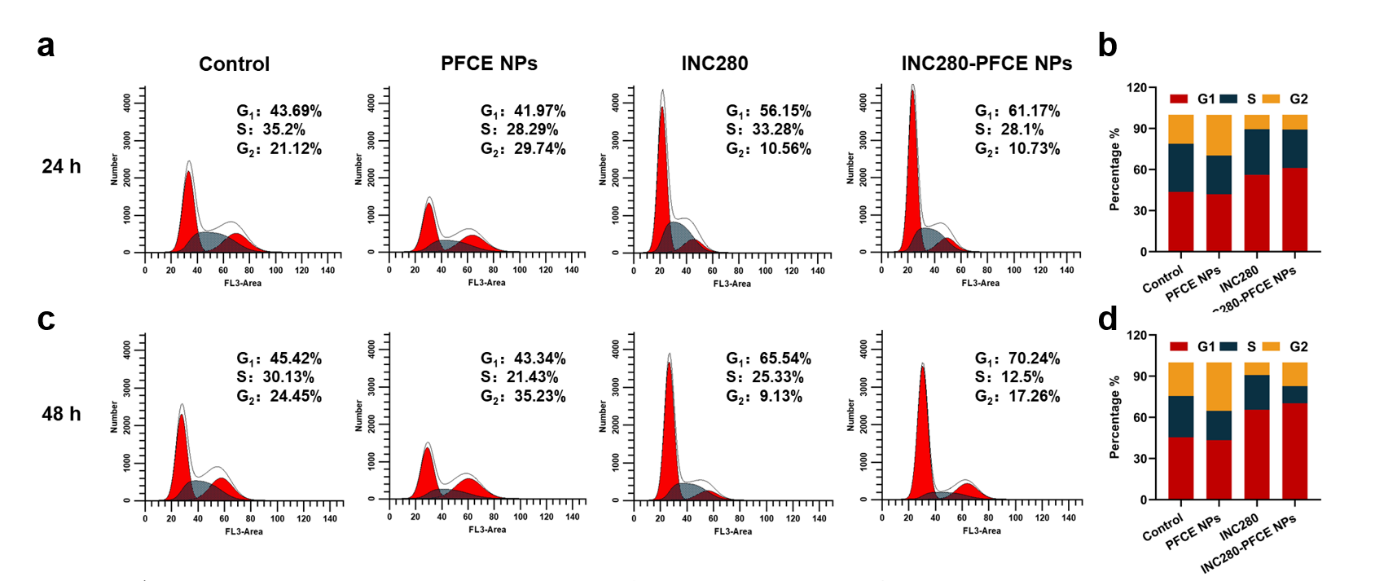


**Supplementary Fig. 12** Detection of EBC-1 cell cycle distribution by flow cytometry. (a) EBC-1 cells were treated with different formulations for 24 h followed by cell cycle analysis using flow cytometry. (b) Quantification of the amounts of EBC-1 cells in G1, S and G2 phase after 24 h shown as bar graphs. (c) EBC-1 cells were treated with different formulations for 48 h followed by cell cycle analysis using flow cytometry. (d) Quantification of the amounts of EBC-1 cells in G1, S and G2 phase after 48 h shown as bar graphs. Data are shown as mean ± standard deviation (n = 3).

**
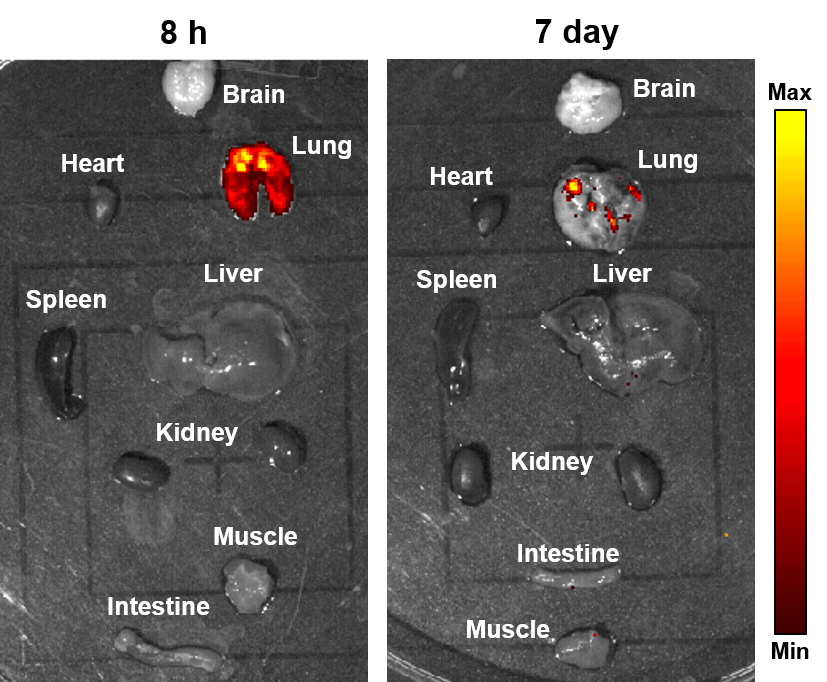
**

**Supplementary Fig. 13** After pulmonary delivery of PFCE NPs, *ex vivo* fluorescence images of the major organs were obtained at 8 h and 7 days.


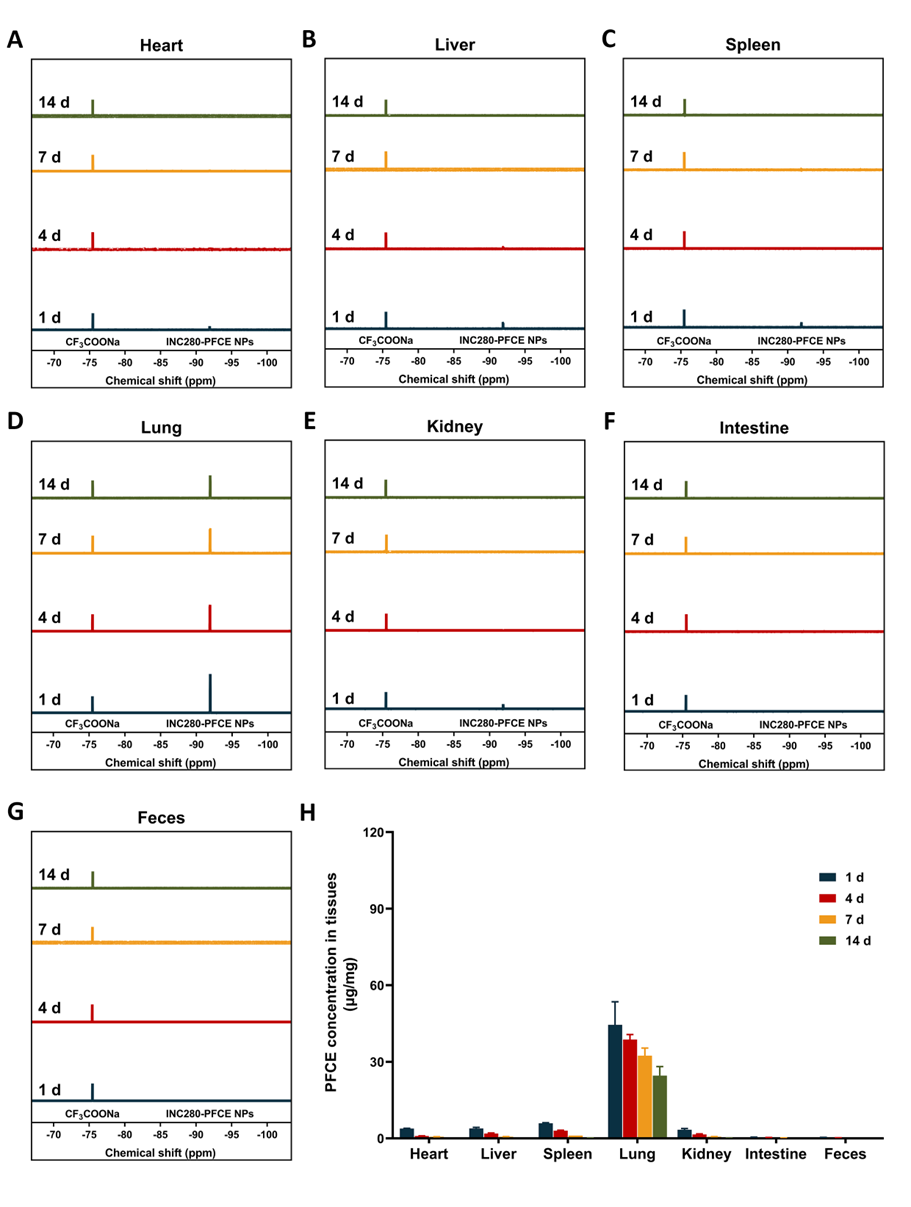


**Supplementary Fig. 14** After different time periods of IT administration with INC280-PFCE NPs, ^19^F-NMR was used to measure PFCE concentration in heart, liver, spleen, lung, kidney, intestine and feces of healthy BALB/c nude mice. CF_3_COONa (-75.4 ppm) was used as an internal reference. Data are presented as mean ± standard deviation (n = 3).

**
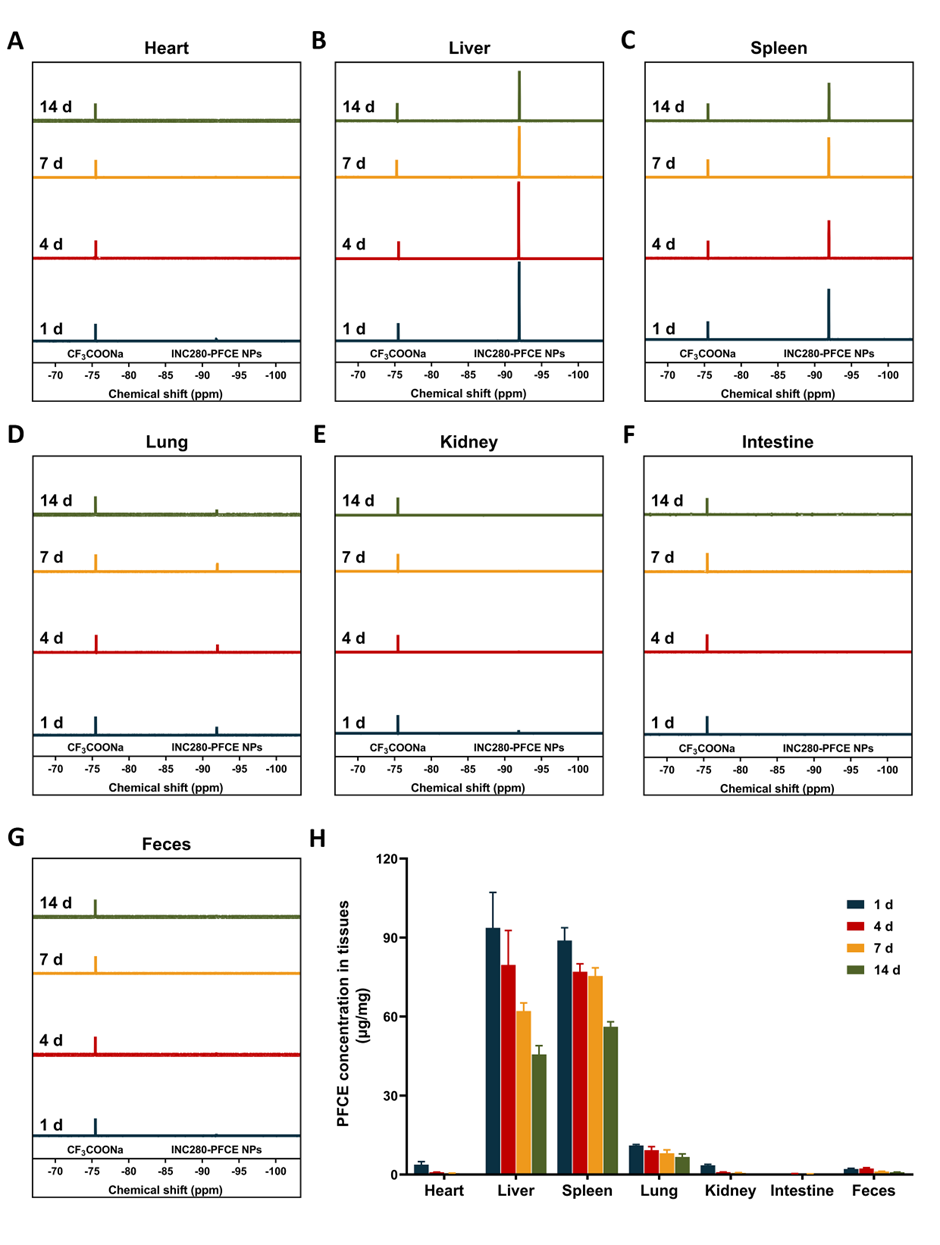
Supplementary Fig. 15** After different time periods of IV administration with INC280-PFCE NPs, ^19^F-NMR was used to measure PFCE concentration in heart, liver, spleen, lung, kidney, intestine and feces of healthy BALB/c nude mice. CF_3_COONa (-75.4 ppm) was used as an internal reference. Data are presented as mean ± standard deviation (n = 3).


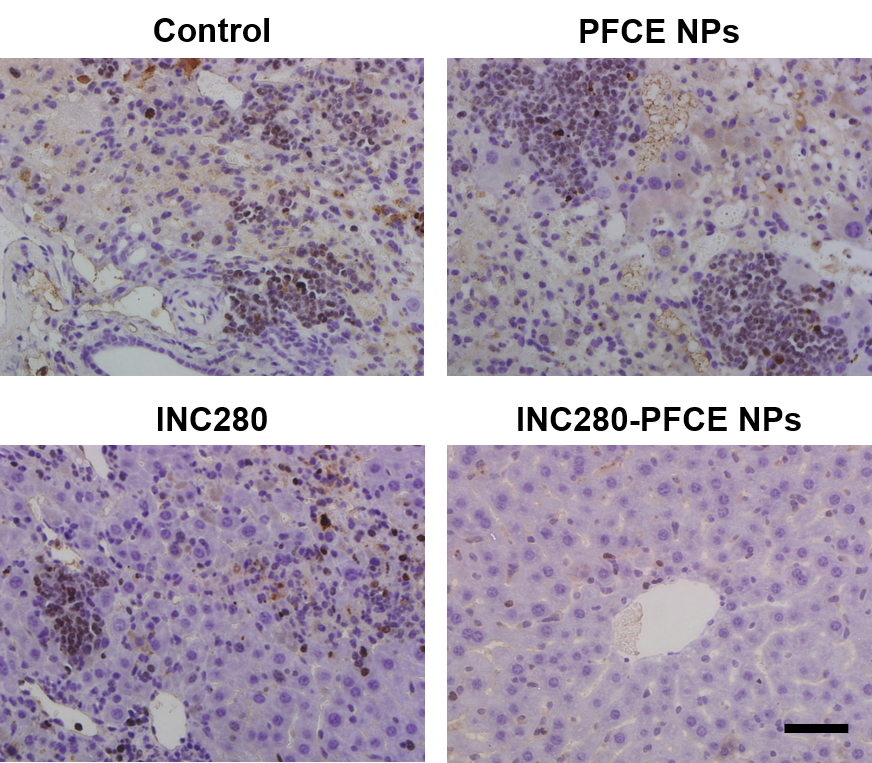


**Supplementary Fig. 16** Ki67 staining of livers harvested from NSCLC liver metastasis model mice after different treatments (scale bar = 100 µm).

**
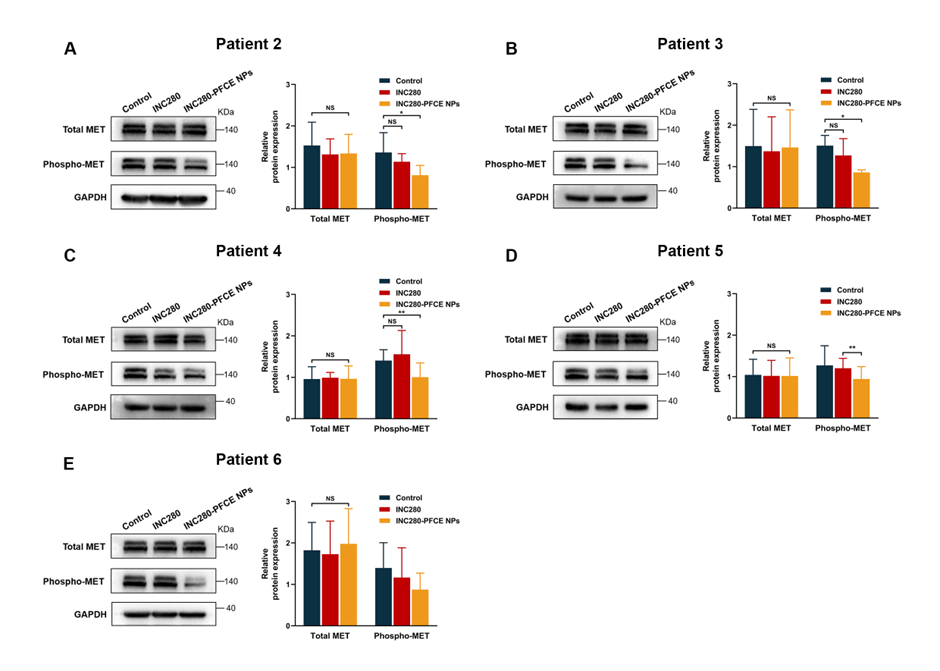
**

**Supplementary Fig. 17** After the PDTFs were processed for 48 h, the expression of total MET and phospho-MET was verified and quantified by western blot. GAPDH was selected as the internal control. Data are presented as mean ± standard deviation (n = 6). * *P* < 0.05 and ** *P* < 0.01.


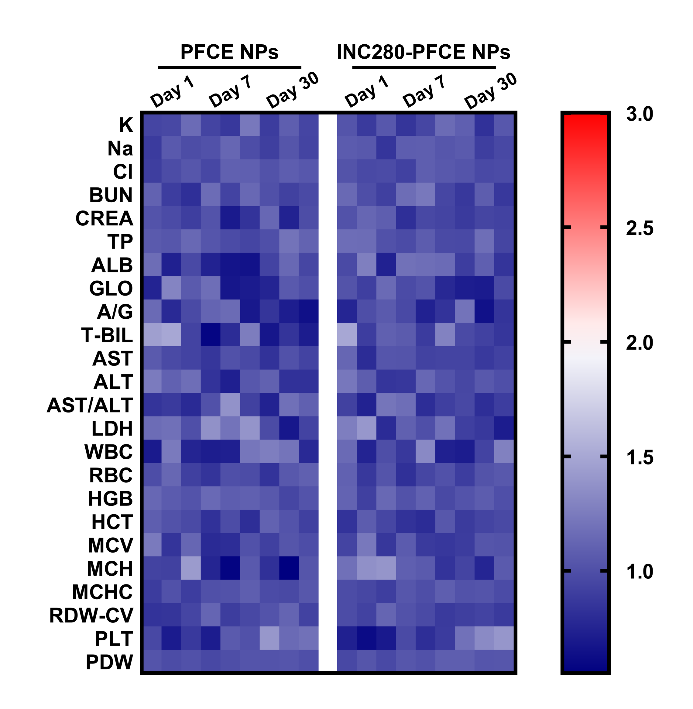


**Supplementary Fig. 18** After pulmonary delivery of PFCE NPs or INC280-PFCE NPs, blood biochemistry and hematological parameters of healthy mice were measured. The study was conducted on days 1, 7 and 30 after administration (n = 3).


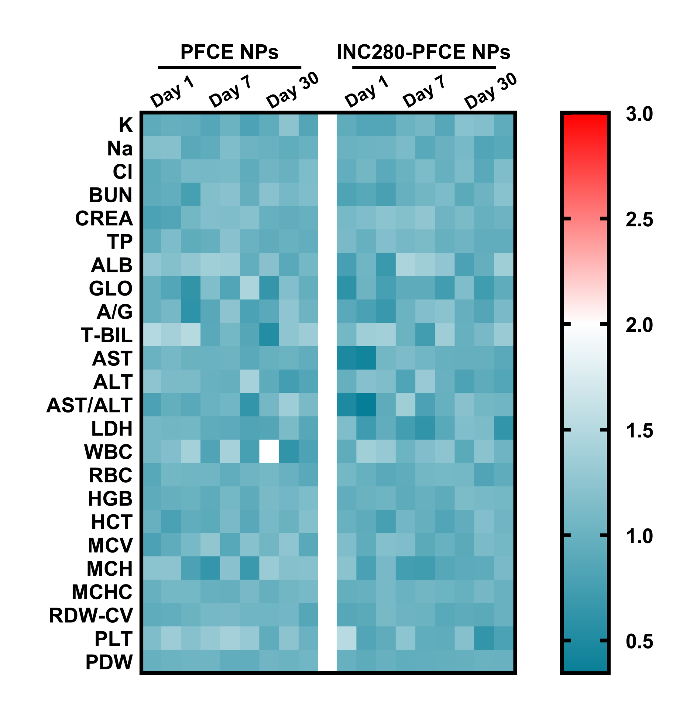


**Supplementary Fig. 19** After intravenous (IV) delivery of PFCE NPs or INC280-PFCE NPs, blood biochemistry and hematological parameters of healthy mice were measured. The study was conducted on days 1, 7 and 30 after administration (n = 3).


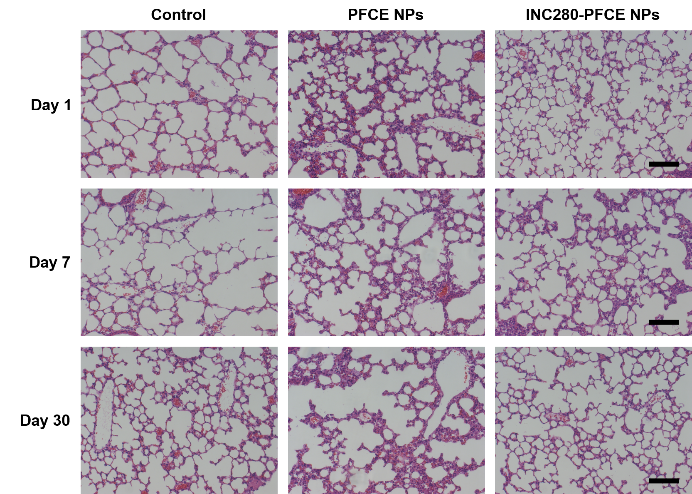


**Supplementary Fig. 20** Effects of pulmonary delivery of PFCE NPs or INC280-PFCE NPs on lung damage. Healthy mice were administered PFCE NPs or INC280-PFCE NPs by pulmonary delivery (n = 3). On days 1, 7 and 30 posttreatment, H&E staining of the lungs was performed. The scale bars are 100 µm.


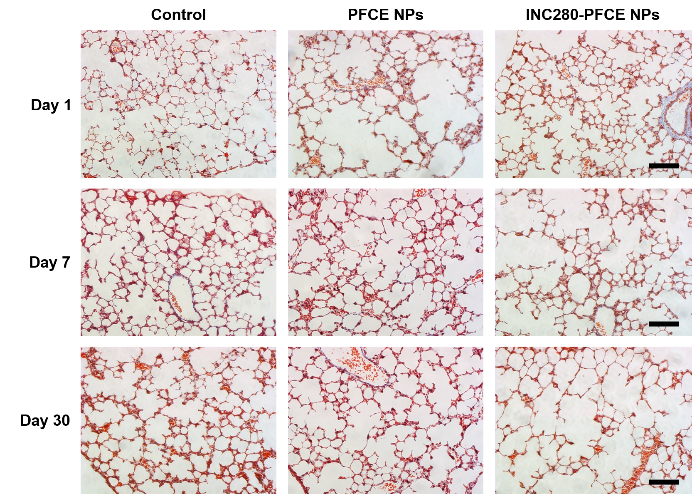


**Supplementary Fig. 21** Effects of pulmonary delivery of PFCE NPs or INC280-PFCE NPs on lung damage. Healthy mice were administered PFCE NPs or INC280-PFCE NPs by pulmonary delivery (n = 3). On days 1, 7 and 30 posttreatment, Masson trichrome staining of the lungs was performed. The scale bars are 100 µm.


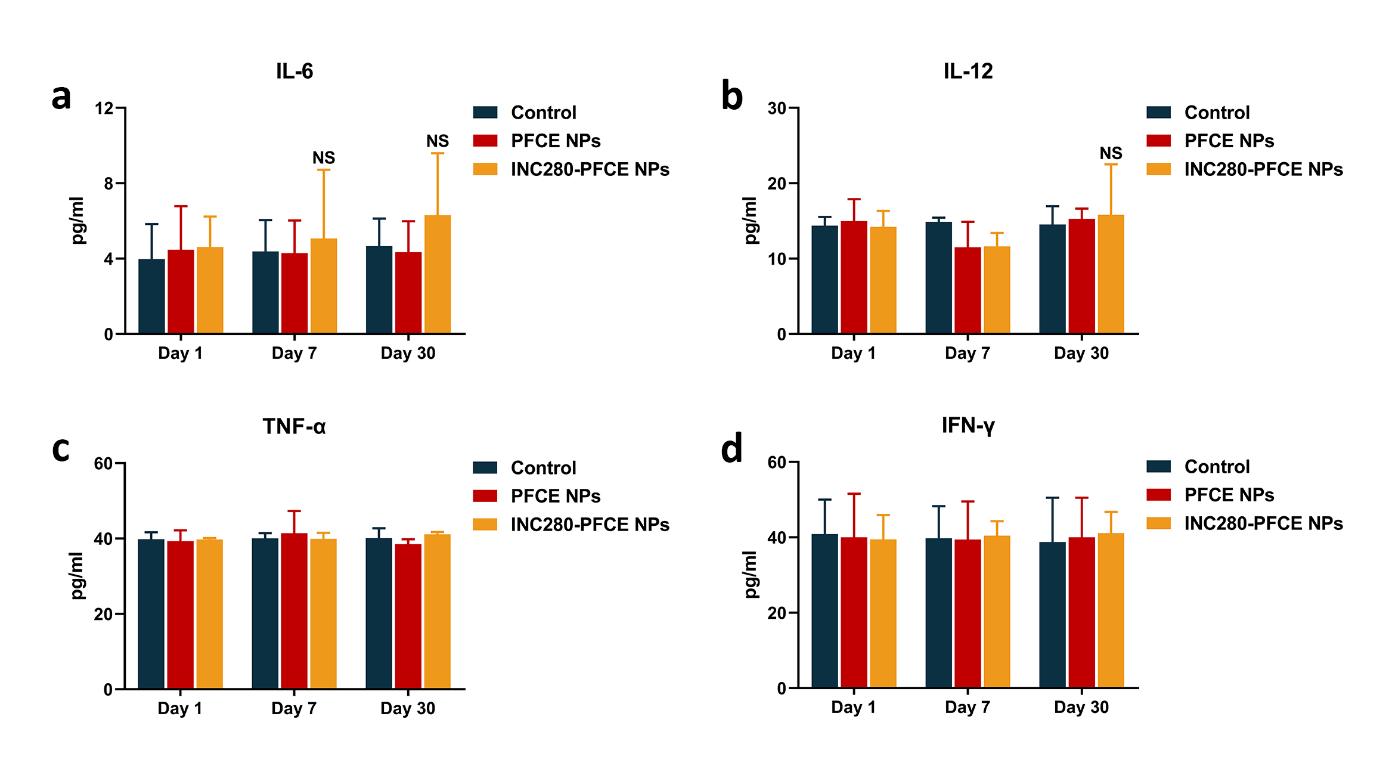


**Supplementary Fig. 22** Effects of pulmonary delivery of PFCE NPs or INC280-PFCE NPs on lung damage. Concentrations of IL-6, IL-12, TNF-α and IFN-γ in the supernatants of the lungs measured by ELISA. The results are shown as mean ± standard deviation (n = 3).


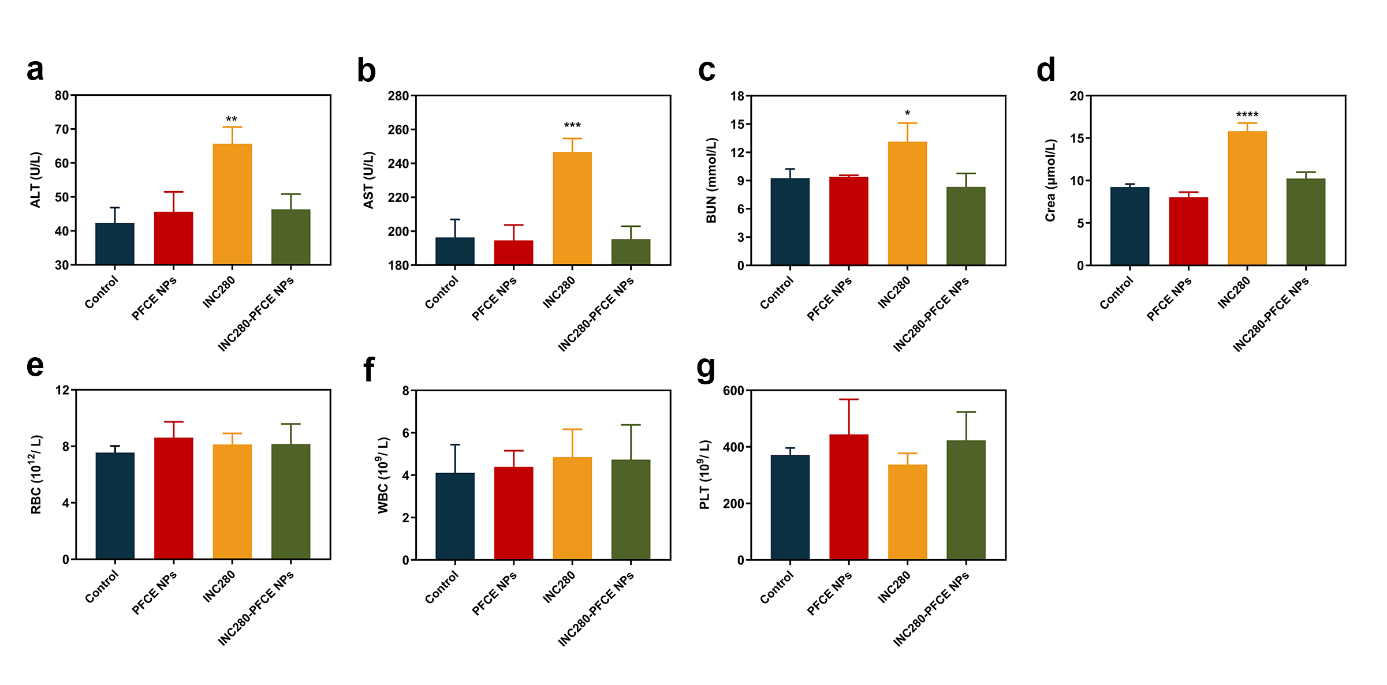


**Supplementary Fig. 23** After 14 days of treatment, (a-b) hepatotoxicity (ALT and AST), (c-d) nephrotoxicity (BUN and Crea), and (e-f) hematological parameters of RBC, WBC and PLT from orthotopic NSCLC model mice treated with different formulations (n = 3). The results are shown as mean ± standard deviation (n = 3), * *P* < 0.05, ** *P* < 0.01, *** *P* < 0.001 and **** *P* < 0.0001.


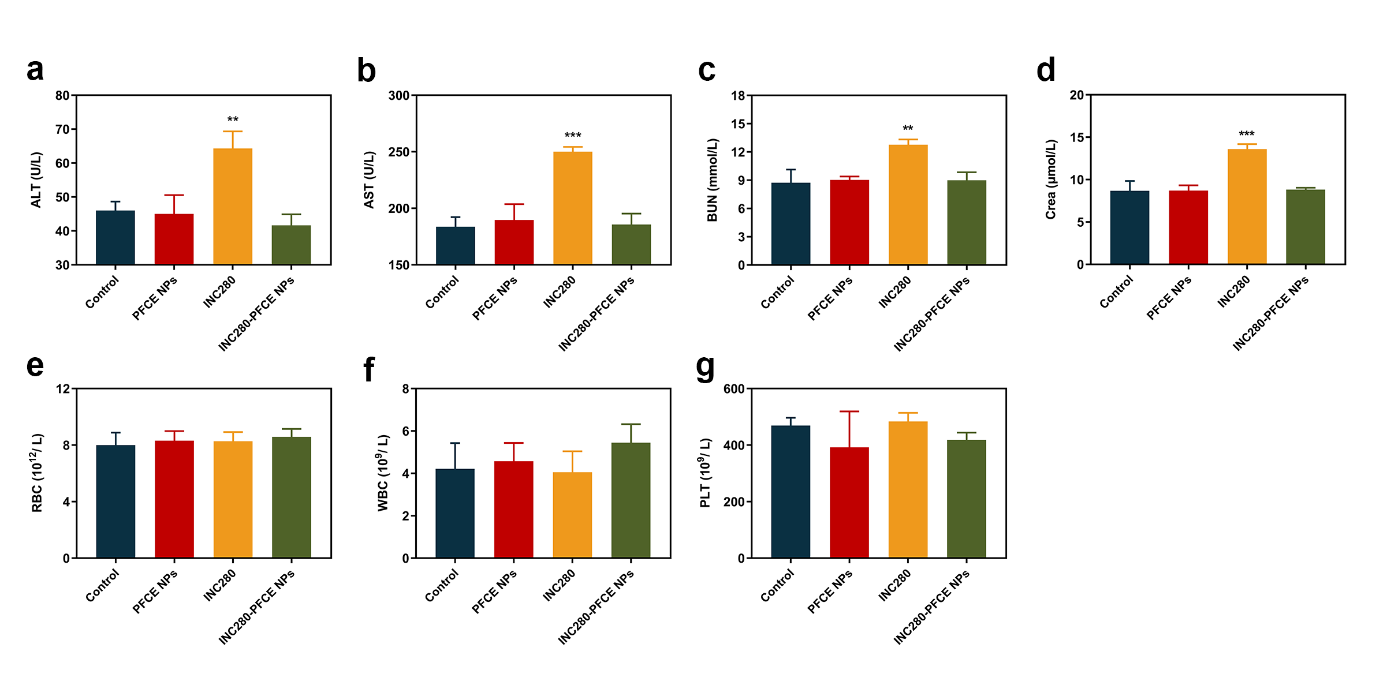


**Supplementary Fig. 24** After 14 days of treatment, (a-b) hepatotoxicity (ALT and AST), (c-d) nephrotoxicity (BUN and Crea), and (e-f) hematological parameters of RBC, WBC, and PLT from NSCLC liver metastasis model mice treated with different formulations (n = 3). The results are shown as mean ± standard deviation (n = 3), ** *P* < 0.01 and *** *P* < 0.001.


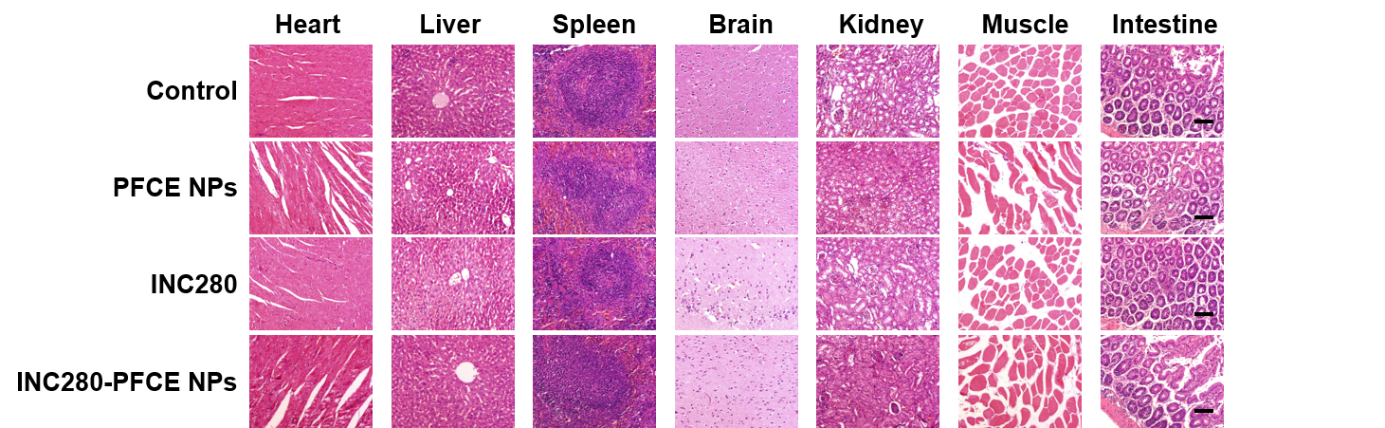


**Supplementary Fig. 25** After 14 days of treatment, H&E staining was performed on the major organs (heart, liver, spleen, brain, kidney, muscle and intestine) of orthotopic NSCLC model mice. Free INC280 was administered orally, and other groups received the appropriate formulation *via* the IT route.

**Supplementary Table 1.** **The** **particle size of INC280-PFCE NPs.**

| Time (weeks) | 4°C | 25°C | 37°C |
| --- | --- | --- | --- |
| 1 | 113.4 ± 4.59 | 105.57 ± 3.36 | 117.7 ± 3.64 |
| 2 | 121.17 ± 1.22 | 99.36 ± 11.42 | 125.5 ± 3.58 |
| 3 | 122.6 ± 2.36 | 103.68 ± 6.48 | 122.13 ±7.44 |
| 4 | 110.06 ± 13.57 | 121.83 ± 2.97^*^ | 115.5 ± 5.07 |
| 5 | 101.06 ± 11.89 | 128.17 ± 4.23^**^ | 138.27 ± 5.75^##^ |
| 6 | 110.07 ± 9.12 | 127.13 ± 7.51^**^ | 134.6 ± 3.03^#^ |

Two-way ANOVA for statistical tests (n = 3). Compared with the size of INC280-PFCE NPs at 25°C for 1 week, * *P* < 0.05, ** *P* < 0.01; Compared with the size of INC280-PFCE NPs at 37°C for 1 week, ^#^ *P* < 0.05, ^##^ *P* < 0.01. Data are shown as mean ± standard deviation.

**Supplementary Table 2. The IC_50_ values of free INC280 and INC280-PFCE NPs.**

| Formulation | Cell | Mean IC_50_ ± SD  （24 h, nM） | Mean IC_50_ ± SD  （48 h, nM） | Mean IC_50_ ± SD  （72 h, nM） |
| --- | --- | --- | --- | --- |
| INC280 | EBC-1 | 1140 ± 0.442 | 61.53 ± 0.326 | 2.053 ± 0.088 |
| INC280-PFCE NPs |  | 719.2 ± 0.456 | 22.2 ± 0.479 | 1.623 ± 0.090 |

The IC_50_ values of free INC280 and INC280-PFCE NPs in EBC-1 cells at 24 h, 48 h and 72 h. Data are shown as the mean ± standard deviation.

**Supplementary Table 3. Patient characteristics and pathological information of all the resected samples.**

| **Sample ID** | **Sex** | **Age** | **Histology** | **Tumor origin** | **TNM stage** |
| --- | --- | --- | --- | --- | --- |
| Patient 1 | Male | 68 | Adenocarcinoma | Primary | T1N0M0 |
| Patient 2 | Female | 65 | Adenocarcinoma | Primary | T1N0M0 |
| Patient 3 | Male | 62 | Sarcomatoid carcinoma | Primary | T2N0M0 |
| Patient 4 | Female | 59 | Adenocarcinoma | Primary | T1N0M0 |
| Patient 5 | Female | 62 | Adenocarcinoma | Primary | T1N0M0 |
| Patient 6 | Female | 61 | Adenocarcinoma | Primary | T1N1M0 |
