# Supplementary material for: Single low-dose INC280-loaded theranostic nanoparticles achieve multirooted delivery for MET-targeted primary and liver metastatic NSCLC
Source: Mol Cancer. 2022 Dec 1;21:212. doi: 10.1186/s12943-022-01681-y (PMC9717478; doi:10.1186/s12943-022-01681-y)
Supplement: Supplementary file 2 — Additional file 2: Supplementary materials and methods. [file 12943_2022_1681_MOESM2_ESM.docx]

**Supplementary Materials and Methods**

**Materials**

INC280 was purchased from GlpBio (USA). High-performance liquid chromatography (HPLC)-grade chloroform and methanol were obtained from Sigma‒Aldrich (Alabama, USA). Phospholipids (lecithin 95% PC and cholesterol) and 1,2-dipalmitoyl-sn-glycero-3-phosphoethanolamine-N-(lissaminerhodamine B sulfonyl) (ammonium salt) (16:0 Liss Rhod PE) were obtained from Avanti Polar Lipids, Inc. (Alabama, USA). Glycerin was acquired from Aldrich Chemical Co. (WI, USA). PFCE (C_10_F_20_O_5_) was purchased from Exfluor Research Corporation (Round Rock, TX, USA). Spectra/Por membranes (Cellulose MWCO: 20 kDa) (Spectrum Medical Industries, Inc., CA, USA) was used for dialysis.

**Synthesis of INC280-PFCE NPs**

INC280, phospholipids (lecithin 95% PC and cholesterol) and 16:0 Liss Rhod PE (10:89.9:0.07 molar ratio) were dissolved in the organic phase comprising chloroform and methanol. Removal of solvent was under reduced pressure (474 bar, 45°C, 200 rpm). The phospholipid film was subsequently dried in a vacuum oven overnight (37°C). PFCE (C_10_F_20_O_5_), glycerin and deionized water were added into the mixture under stirring for 30 min. The crude suspension underwent a precooled high-pressure homogenization process in an M110P Microfluidics with continuous pressurization (20,000 psi) to pass through the microfluidics device 10 times. Finally, the as-prepared INC280-PFCE NPs were dialyzed using a 20-kDa MWCO cellulosic membrane under clean and sterile conditions and kept in nitrogen-sealed vials at 4°C.

**Characterization of INC280-PFCE NPs**

The hydrodynamic size and zeta potentials of the NPs in aqueous medium were determined by DLS (Zetasizer Nano ZS90, Malvern Instruments, UK). To evaluate the effect of temperature on the stability of nanoparticles, the size of INC280-PFCE NPs in deionized water was monitored by DLS after storage at 4°C, 25°C and 37°C. In addition, the size stability test of INC280-PFCE NPs was also performed in fetal bovine serum (FBS) and culture medium at 4°C. The morphology of the INC280-PFCE NPs was observed and photographed by transmission electron microscopy (TEM) (HT7800, Hitachi, Japan). HPLC was performed to analyze the INC280 content in the INC280-PFCE NPs in accordance with the previous descriptions[1]. The encapsulation efficiency (EE) of INC280 was calculated by the following formula: 𝐸𝐸 = 𝑊_𝐸𝑛𝑐𝑎𝑝𝑠𝑢𝑙𝑎𝑡𝑒𝑑_/𝑊_𝐹𝑒𝑑_ × 100%, where 𝑊_𝐸𝑛𝑐𝑎𝑝𝑠𝑢𝑙𝑎𝑡𝑒𝑑_ is the weight of INC280 incorporated into the NPs, and 𝑊_𝐹𝑒𝑑_ represents the weight of INC280 initially fed for encapsulation. To further assess the ^19^F-MRI properties of the INC280-PFCE NPs, the phantoms were diluted to different concentrations and assessed with a 9.4 T MR scanner (BioSpec 94/20 USR; Bruker BioSpin, Germany). ^19^F NMR of the INC280-PFCE NPs was conducted on a 9.4T Bruker instrument (564 MHz) with a BBFO cryoprobe using a delay time of 18 µs and 32 scans. INC280-PFCE NPs were dissolved in D_2_O (Sigma–Aldrich, Germany). 0.1% (w/v) CF3COONa (Macklin, China) was used as a reference [2].

**Cellular uptake assay**

EBC-1 cells were seeded in confocal dishes (1×10^5^ cells/well) and grown until they reached 60~80% confluency. At 4 h, 12 h and 24 h after incubation with rhodamine-labeled INC280-PFCE NPs (10 nM INC280 and 300 nM PFCE) at 37°C, EBC-1 cells were rinsed and fixed. And at 4 h, 12 h, 24 h and 48 h after incubation with rhodamine-labeled PFCE NPs (300 nM PFCE) at 37°C, then EBC-1 cells were also rinsed and then fixed. In addition, the nuclei staining was performed with 4′,6-diamidino-2-phenylindole (DAPI). Finally, EBC-1 cells were imaged by confocal laser scanning microscopy (CLSM) (Nikon, Japan). Quantification of the mean fluorescence intensity (MFI) was performed by ImageJ.

**Cell viability assay**

EBC-1 cells were seeded in 96-well plates (5×10^3^ cells/well) and grown overnight. Then, EBC-1 cells were exposed to PFCE NPs (0.01 to 1000 nM PFCE) at various concentration and incubated for 24 h, 48 h and 72 h. In addition, EBC-1 cells were exposed to free INC280 (0.1 to 1000 nM INC280) or INC280-PFCE NPs (0.1 to 1000 nM INC280) and incubated for 24 h, 48 h and 72 h. Viability of EBC-1 cells was assessed by MTT assay on the basis of a previously described protocol[3]. The absorbance value was determined by a microplate reader at 570 nm (BioTek, USA).

**Cell apoptosis assay**

EBC-1 cells were seeded at a density of 3×10^5^ cells/well in 6-well plates and cultivated with different reagents for 24, 48 and 72 h. The group included Control, PFCE NPs (300 nM PFCE), INC280 (10 nM INC280) and INC280-PFCE NPs (10 nM INC280 and 300 nM PFCE). The groups of free INC280 and INC280-PFCE NPs had the same concentration of INC280 (10 nM). The groups of PFCE NPs and INC280-PFCE NPs had the same concentration of PFCE (300 nM). EBC-1 cells were then harvested, washed and collected by centrifugation (1000 rpm, 5 min). Subsequently, 100 µL of binding buffer containing 5 µL of Annexin V-FITC and 5 µL of propidium iodide (PI) were mixed with the cell suspension for 15 min and then were subjected to analysis of the apoptotic rate (%) with a flow cytometer (Beckman Coulter, California, USA). Apoptotic cells could also be detected by TUNEL assay with the *In-Situ* Cell Death Detection Kit (Roche, Branchburg, NJ, USA) in terms of the previous descriptions[4]. The TUNEL-positive cells were detected by CLSM.

**Cell cycle assay**

To further evaluate the cell cycle transition, EBC-1 cells (4×10^5^) were subjected to different treatments for 24, 48 and 72 h. The experimental group included Control, PFCE NPs (300 nM PFCE), INC280 (10 nM INC280) and INC280-PFCE NPs (10 nM INC280 and 300 nM PFCE). The free INC280 and INC280-PFCE NPs groups had the same concentration of INC280 (10 nM). The PFCE NPs and INC280-PFCE NPs groups had the same concentration of PFCE (300 nM). Then, the cells were trypsinized and washed twice. The cell suspensions were fixed in prechilled 75% ethanol at 4°C overnight and subsequently stained with PI (50 µg/mL) for 15 min away from light. EBC-1 cell cycle distribution was analyzed by flow cytometry (Beckman Coulter, California, USA). Quantitative analysis was performed with ModFit LT 4.1 (VSH, USA).

**Western blot**

Protein expression levels were analyzed by western blot as previously described [5]. EBC-1 cells were treated with PFCE NPs (30 nM PFCE), free INC280 (1 nM INC280), and INC280-PFCE NPs (1 nM INC280 and 30 nM PFCE) for 4 h. The groups of free INC280 and INC280-PFCE NPs had the same concentration of INC280 (1 nM). And the groups of PFCE NPs and INC280-PFCE NPs had the same concentration of PFCE (30 nM). After being subjected to different treatments, lysis of EBC-1 cells or human lung cancer tissues were in prepared RIPA (ThermoFisher) containing phosphatase (ThermoFisher) and proteinase (Roche) inhibitors in a cold bath. Samples with equal amounts of protein were determined by a BCA Protein Assay Kit. Then, the proteins were separated by sodium dodecyl sulfate‒polyacrylamide gel electrophoresis (SDS‒PAGE). The bands were electrophoretically transferred to a nitrocellulose membrane and blocked with 5% nonfat milk in TBST. The membranes were incubated with primary rabbit polyclonal antibodies against total MET cat. #8198s with a dilution of 1:1000; phospho-MET (Tyr1234/1235) cat. #3077 with a dilution of 1:1000; total ERK1/2 cat. #4695 with a dilution of 1:1000; phospho-ERK (Thr202/Tyr204) cat. #4370 with a dilution of 1:1000; total AKT cat. #4691 with a dilution of 1:1000; and phospho-AKT (Ser473) cat. #4060 at a dilution of 1:1000, all of which were from Cell Signaling Technology (CST). Finally, the membranes were incubated with secondary antibody and imaged by an ECL Western Blotting detection system (BD).

**Biodistribution studies**

*Ex vivo* fluorescence imaging (excitation/emission, 500~550/575~650 nm) was conducted on the orthotopic NSCLC model animals. Rhodamine-labeled PFCE NPs or INC280-PFCE NPs were given via IT instillation (50 µL, n = 3). *Ex vivo* fluorescence imaging of the major organs and tumors were performed and analyzed on an IVIS spectrum imaging system (PerkinElmer, Waltham, MA, USA) at 8 h and 7 days after administration. Major organs and feces of healthy BALB/c nude mice were collected for analyzing PFCE contents by ^19^F-NMR. Briefly, mice were administered with INC280-PFCE NPs *via* IT instillation (50 μL) or IV injection (100 μL). At the days of 1st, 4th, 7th, and 14th posttreatment, mice were sacrificed. Major organs (heart, liver, spleen, lung, kidney and intestine) and feces of the mice were collected, lysed in prepared RIPA buffer and centrifuged (13200 g, 10 min). Then, the supernatants were dissolved in D_2_O and 0.1% (w/v) CF_3_COONa was added as a reference. ^19^F-NMR experiments were conducted on a 9.4T Bruker instrument (376 MHz) with a BBFO cryoprobe using a delay time of 2 µs and 32 scans. The INC280 contents in the tumors and major organs were determined by HPLC. Briefly, mice with orthotopic NSCLC tumors were given free INC280 via oral administration and INC280-PFCE NPs via IT instillation at a dose of 5 mg/kg INC280 (n = 3). After 24 h of administration, the mice were executed. Then, the excised tumors and major organs were measured their mass for HPLC analysis as described previously [6].

**NSCLC** **liver metastasis model and *in vivo* ^19^F-MRI**

The NSCLC liver metastasis model was established by injecting an EBC-1 cell suspension into the tail vein (100 μL, 10^7^ cells/mL). After 2~3 weeks of following injection, NSCLC liver metastasis formation was identified by ^1^H-MRI. The mice with NSCLC liver metastases were anesthetized and performed MRI on a 9.4 T MR scanner following the procedures as detailed above. INC280-PFCE NPs were given by tail vein injection in a volume of 100 μL (n = 3), and *in vivo* ^19^F-MRI was performed at 4~72 h and 4 days. ^1^H-MR images were obtained with a T2-RARE sequence with the following parameters: TR/TE = 3000/40 msec, RARE factor = 10, NA = 8, ST = 1.0 mm, matrix = 256 × 256, FOV = 38.4 × 38.4 mm^2^. The corresponding ^19^F-MR images were acquired using the RARE sequence (TR/TE = 2000/100 msec, RARE factor = 32, NA = 128, ST = 3.0 mm, matrix = 64×64, FOV = 38.4×38.4 mm^2^). Signal quantification is analyzed by a reference phantom containing 10.37 mg/mL PFCE NPs. Processing of the images and ^19^F-MR signal quantification were consistent with the above descriptions.

**Biosafety evaluation *in vivo***

In brief, healthy female BALB/c nude mice (5~6 weeks) were intratracheally (50 µL) or intravenously (100 µL) administered with INC280-PFCE NPs or PFCE NPs. After 1, 7 and 30 days of administration, the mice were euthanized. The lungs were acquired for H&E staining and Masson trichrome staining. In addition, ELISA was conducted to assess the cytokines levels of IL-6, IL-12, TNF-α and IFN-γ. Briefly, freshly excised mouse lungs were lysed for 30 min with prepared RIPA buffer and centrifuged (12,000 rpm, 10 min). In accordance with the manufacturer’s protocol, the concentration of IL-6, IL-12, TNF-α and IFN-γ in the supernatants were detected by ELISA kits (BioLegend, USA). To further demonstrate the biological toxicity of the posttreatment groups, we performed blood cell analysis and blood biochemical analysis on the orthotopic NSCLC and NSCLC liver metastasis model mice. On day 14 posttreatment, complete blood specimens were collected for red blood cells (RBCs), white blood cells (WBCs) and platelets (PLTs) analyses. Blood serum was collected for AST, ALT, BUN and Crea analysis. Finally, the major organs of the mice with orthotopic NSCLC tumors were collected for H&E staining.

**References**

1. Xia, J., S. Ma, X. Zhu, C. Chen, R. Zhang, Z. Cao, et al., Versatile ginsenoside Rg3 liposomes inhibit tumor metastasis by capturing circulating tumor cells and destroying metastatic niches. Sci Adv. 2022;8:eabj1262.

2. Janjic, J.M., M. Srinivas, D.K. Kadayakkara, and E.T. Ahrens, Self-delivering nanoemulsions for dual fluorine-19 MRI and fluorescence detection. J Am Chem Soc. 2008;130:2832-41.

3. Shamay, Y., M. Elkabets, H. Li, J. Shah, S. Brook, F. Wang, et al., P-selectin is a nanotherapeutic delivery target in the tumor microenvironment. Sci Transl Med. 2016;8:345ra87.

4. Grasl-Kraupp, B., B. Ruttkay-Nedecky, H. Koudelka, K. Bukowska, W. Bursch, and R. Schulte-Hermann, In situ detection of fragmented DNA (TUNEL assay) fails to discriminate among apoptosis, necrosis, and autolytic cell death: a cautionary note. Hepatology. 1995;21:1465-8.

5. Shimokawa, T., M. Seike, C. Soeno, H. Uesaka, A. Miyanaga, H. Mizutani, et al., Enzastaurin has anti-tumour effects in lung cancers with overexpressed JAK pathway molecules. Br J Cancer. 2012;106:867-75.

6. Tailor, T.D., G. Hanna, P.S. Yarmolenko, M.R. Dreher, A.S. Betof, A.B. Nixon, et al., Effect of pazopanib on tumor microenvironment and liposome delivery. Mol Cancer Ther. 2010;9:1798-808.
